# Effects of exercise on cognitive function in older patients with Alzheimer’s disease: a meta-regression and meta-analysis

**DOI:** 10.3389/fpubh.2026.1793973

**Published:** 2026-04-08

**Authors:** Feng Xu, Yiwan Zhang, Danqing Tan, Yiming He, Qinfei Fei, Kai Xu

**Affiliations:** 1 Department of Physical Education, Shanghai Maritime University, Shanghai, China; 2 School of Sports Science, South China Normal University, Guangzhou, Guangdong, China; 3 School of Economics and Management, Shanghai Maritime University, Shanghai, China; 4 College of Ocean Science and Engineering, Shanghai Maritime University, Shanghai, China; 5 Merchant Marine College, Shanghai Maritime University, Shanghai, China; 6 School of Athletic Performance, Shanghai University of Sports, Shanghai, China

**Keywords:** Alzheimer’s disease, exercise, cognitive function, meta-analysis, meta-regression, older adults, dose-response, physical activity

## Abstract

**Objective:**

This study aimed to systematically evaluate the effects of exercise intervention on cognitive function in AD patients through meta-analysis, specifically investigating the dose–response relationships and moderating effects of various exercise prescription parameters including frequency, single-session duration, weekly total duration, total intervention duration, type, and intensity—on the magnitude of cognitive improvement. The findings are expected to provide scientific and actionable empirical evidence for designing precise and individualized exercise programs.

**Methods:**

Relevant literatures were systematically retrieved from eight databases up to August 2025, including PubMed, Web of Science, Embase, Emcare, Scopus, Cochrane Library, Ebsco, and SPORTDiscus. All included trials were randomized controlled trials (RCTs) involving adults aged 60 years and above. These studies examined the effects of exercise intervention on cognitive function in AD patients, compared with passive control groups without exercise. A multilevel meta-analysis was used to assess the impact of exercise on cognitive function outcomes in AD patients. Additionally, multivariate meta-regression analysis was employed to identify the exercise frequency (sessions per week) that maximizes cognitive improvement, as well as key moderating factors.

**Results:**

Data from 23 studies, involving 1,868 adults, were included. Exercise intervention significantly improved global cognitive function in AD patients (*g* = 0.22, 95% CI: 0.02–0.41, *p* < 0.001). Patient age, single-session duration, and total weekly exercise duration did not significantly affect outcomes (*p* > 0.05). However, the cognitive benefits of exercise were significantly enhanced with increased weekly exercise frequency, with a notable strengthening trend observed when sessions exceeded five per week.

**Conclusion:**

Exercise intervention can effectively improve cognitive function in AD patients. “To optimize intervention outcomes, exercise prescription should consider higher frequency (e.g., more than five sessions per week may be associated with greater cognitive benefits) and the cumulative effect during the initial intervention phase (e.g., sustained beyond 12 weeks may represent a critical window for short-term improvement). In addition, a potential plateau in long-term benefits suggests the need for periodic program adjustments and multimodal exercise strategies. These findings should be interpreted as exploratory dose–response associations rather than definitive clinical prescriptions.

**Systematic review registration:**

https://www.crd.york.ac.uk/prospero/display_record.php?RecordID=1012816, identifier CRD420251012816.

## 1 Introduction

Alzheimer’s disease (AD), a progressive neurodegenerative disorder, has become an increasingly severe global public health challenge (1). A typical clinical feature is that recent memory decline predominates in the early stages, while remote memory remains relatively preserved. As the disease progresses, remote memory is gradually impaired, and in severe cases, patients may forget the names of their family members and even their own identities (2). AD can also lead to a range of serious consequences, including emotional disturbances such as depression, anxiety, agitation, and apathy, as well as significant declines in activities of daily living (ADL) (3, 4). According to the 2021 World Alzheimer Report, ~55 million people worldwide live with dementia, among which AD accounts for 60–70%. This number is projected to rise to 139 million by 2050 (5). As one of the most rapidly aging populations globally, China has over 10 million AD patients, representing a quarter of the world’s total. This imposes substantial medical, economic, and social burdens on healthcare systems, families, and social security (6). Studies indicate that the progression rate of AD varies across populations, with incidence rising significantly among older adults (7). Specifically, among people aged ≥55 years, AD cases increased by 143.88% between 1992 and 2021, with age being the primary driving factor. The risk of developing AD rises exponentially in individuals over 80 years old (8). Concurrently, the prevalence of subclinical AD symptoms, which do not meet full diagnostic criteria, has also increased markedly among the older adults (9). The COVID-19 pandemic has severely disrupted daily life for older adults worldwide and exacerbated pre-existing stressors (10). Prolonged social isolation, lack of family companionship, and increased social pressures may further elevate the risk of developing AD (11). Therefore, more effective approaches are needed to improve cognitive function and quality of life in these patients. Treating AD is a long-term, costly, and often ineffective process, placing substantial economic and psychological burdens on patients’ families and society (12). In terms of pharmacotherapy, there are currently no specific and efficacious treatment regimens available (13).

Non-pharmacological therapies are recognized as safe, relatively low-cost, and scalable interventions (14). Among these, exercise intervention is considered a highly promising approach for the management and prevention of AD, owing to its multiple physiological and psychological benefits, low risk, and ease of implementation (15). Exercise is believed to improve cardiovascular health and mental well-being, such as by alleviating symptoms of anxiety and depression (16). Moreover, physical activity in older adults is positively correlated with cognitive function (17).

AD patients can benefit from various exercise modalities, including resistance and aerobic training. It has been reported that exercise intervention significantly improves cognitive function in AD patients, with combined resistance and aerobic exercise showing the strongest effect (18, 19). By improving vascular health and enhancing neuroplasticity, exercise yields particularly notable cognitive and functional benefits in patients with mild-to-moderate AD, making it easily scalable in community and nursing home settings (20). Mechanistic studies have confirmed that myokines such as IL-6, released by skeletal muscle during exercise, promote anti-inflammatory factor secretion, inhibit Aβ deposition and abnormal tau phosphorylation, upregulate brain-derived neurotrophic factor (BDNF) expression, and enhance hippocampal neurogenesis. These mechanisms help protect neuronal function in AD patients without the adverse effects associated with pharmacotherapy (21).

When evaluating the impact of exercise on cognitive function in AD patients, the Mini-Mental State Examination (MMSE) is a common and effective assessment tool (22). In the present study, we screened eight databases and analyzed the measurement tools for assessing exercise effects on cognition, including the MMSE (23) and the cognitive subscale of Alzheimer’s Disease Assessment Scale (ADAS-cog) (24).

Therefore, overcoming the limitations of prior studies and meeting the clinical need for precise exercise prescriptions, this study aimed to systematically evaluate the effect of exercise intervention on cognitive function in AD patients through meta-analysis. Furthermore, it sought to explore in depth the dose–response relationships and moderating effects of different exercise prescription parameters including frequency, single-session duration, total intervention duration, type, and intensity—on cognitive outcomes, in order to provide empirical evidence for developing precise and individualized exercise programs.

## 2 Methods

### 2.1 Protocol and registration

The protocol for this systematic review and meta-analysis was prospectively registered with the International Prospective Register of Systematic Reviews (CRD 420251012816). The Preferred Reporting Items for Systematic Reviews and Meta-Analyses (PRISMA) Statement 2020 was followed (25).

### 2.2 Inclusion criteria

The inclusion criteria were developed according to the PICOS framework:

1. *Participants*: Adults aged ≥ 60 years, with no restrictions regarding the baseline clinical diagnostic status of AD (including preclinical stage, mild cognitive impairment, and dementia stage). This allowed a comprehensive evaluation of the preventive and therapeutic effects of exercise across different stages of AD.
2. *Interventions*: The experimental group was required to undergo a systematic exercise intervention. Exercise modalities included, but were not limited to: aerobic exercise (e.g., walking, running, swimming), resistance training (e.g., machine-based training, elastic band training), balance training (e.g., Tai Chi, balance board training), mind–body exercise (e.g., yoga, Qigong), and multimodal exercise (combining two or more exercise types). No mandatory restrictions were placed on exercise intensity, frequency, or single-session duration.
3. *Controls*: The control group received non-exercise interventions, which took the following specific forms: blank control (no intervention), usual care (maintenance of existing medical regimens), wait-list control (delayed intervention), and active control (participation in passive activities unrelated to exercise, such as watching educational videos or listening to music).
4. *Outcomes*: Studies were required to use validated standardized cognitive assessment tools. These included, but were not limited to: the psychomotor reaction time test, Montreal Cognitive Assessment (MoCA), MMSE, Cambridge Cognitive Examination (CAMCOG), Clock Drawing Test (CDT), Verbal Fluency Test, and Trail Making Test Part A. All listed tools are recognized for their good reliability and validity in assessing cognitive function.
5. *Study design*: Only experimental designs featuring a control group and pre- and post-intervention cognitive function assessments were included. Eligible designs encompassed randomized controlled trials (RCTs), non-randomized controlled trials, and similar designs.

Exclusion criteria:

1. Non-original studies, such as systematic reviews, meta-analyses, narrative reviews, and commentaries;
2. Studies that failed to report complete effect size data or key study information, including conference abstracts, dissertations, and study protocols (26);
3. Clinical trials from which valid data could not be extracted or that lacked baseline data;
4. Interventions incorporating other components that could significantly confound cognitive function assessment (e.g., combined cognitive training, pharmacotherapy, etc.);
5. Studies published in languages other than Chinese or English for which full texts were unavailable (27).

### 2.3 Information sources and search strategy

A systematic search was conducted across eight databases in August 2025: Web of Science, PubMed, Embase, Cochrane Library, Ebsco, Scopus, SPORTDiscus, and PsycInfo. The detailed search strategies for each database are provided in Appendix S1 of the Electronic Supplementary material (ESM). To identify additional relevant studies, the reference lists of eligible articles, as well as relevant topic-focused systematic reviews and meta-analyses, were also searched.

### 2.4 Study selection

Two researchers (DT and YH) independently assessed the eligibility of studies against the inclusion and exclusion criteria, documenting the process using a standardized data extraction form. Any discrepancies were resolved through discussion with a third researcher (FX) until consensus was reached, thereby confirming the final set of included studies. To minimize the risk of omitting relevant literature, the reference lists of all included studies were also screened.

### 2.5 Data collection process and data extraction

Two authors (DT and YH) performed the initial data extraction, and a third author (FX) verified the extracted data. A standardized data extraction form was specifically developed to systematically record information from each study. The form primarily included the following elements:

1. First author’s name and year of publication;
2. Article title;
3. Sample characteristics (e.g., sample size, mean age, clinical AD diagnosis, disease severity), exercise type and frequency, and specific intervention details (e.g., exercise type, frequency [sessions/week], total weekly exercise duration [minutes], total intervention duration [weeks], and single-session duration [minutes]);
4. Methodological information (e.g., potential risk of bias, diagnostic tools, and symptom assessment methods);
5. Main outcomes (e.g., pre- to post-intervention changes in AD symptom scores);
6. Study design (including presence of an independent control group and use of a crossover design).

### 2.6 Risk of bias assessment

**Figure 1:**
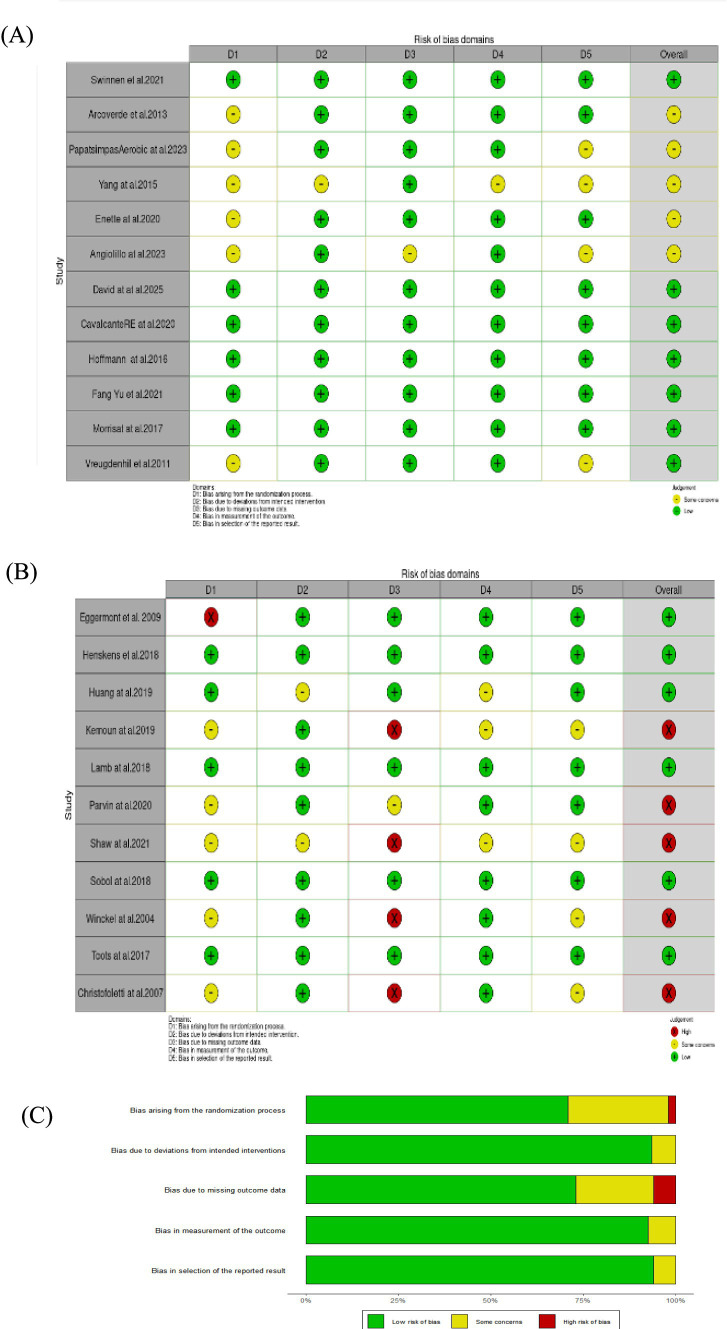
Risk of bias assessment results using the ROB 2.0 tool. **(A, B)** Traffic light plot summarizing the risk of bias judgments across domains for each included study. **(C)** Bar chart displaying the proportion of studies with low, some concerns, and high risk of bias across each domain.

Two researchers (DT and YH) independently assessed the risk of bias using the methods recommended by the Cochrane Collaboration. Discrepancies were resolved by a senior researcher (FX). The full risk-of-bias assessment is provided in Appendix S2 of the Electronic Supplementary material (ESM). Prior to the assessment, the researchers familiarized themselves with the Cochrane Risk of Bias Tool Version 2.0 (ROB 2.0) (28) through pilot testing. Relevant traffic light plots and bar plots were generated using the ROB 2.0 tool (Figure 1).

### 2.7 Assessment of certainty of evidence

Two researchers (DT and YH) independently assessed the certainty of evidence using the Grading of Recommendations, Assessment, Development and Evaluations (GRADE) framework (29). Each researcher evaluated the included studies separately, and the certainty of evidence was classified into four levels: high, moderate, low, and very low. As all included studies were randomized controlled trials (RCTs), the initial certainty rating was set as high. The final certainty rating was determined based on confidence in the effect estimates and was adjusted by considering limitations in study design and implementation, inconsistency of results, indirectness of evidence, and imprecision of outcomes. The certainty of evidence was downgraded if any of the following conditions were met: (1) *I*^2^ > 50%, indicating substantial heterogeneity and insufficient consistency (30); (2) significant differences in population characteristics, outcome measures, or interventions across studies, indicating indirectness; (3) a total sample size of less than 800 for a given outcome analysis, resulting in imprecision (31); or (4) a significant publication bias indicated by Egger’s test. Conversely, the certainty of evidence could be upgraded by one level if any of the following criteria were satisfied: (1) a large effect size (standardized mean difference [SMD] > 0.8); (2) evidence of a clear dose–response relationship; or (3) confirmation that plausible residual confounding factors would reduce, rather than increase, the observed effect. It should be noted that all meta-regression analyses (both univariate and multivariate) were considered exploratory in nature, intended to generate hypotheses rather than to establish causal relationships. Accordingly, these findings should be interpreted as preliminary indications of dose–response associations rather than definitive conclusions (32).

### 2.8 Data synthesis and analysis

Quantitative synthesis and analysis were performed using the metafor and rms packages in R software; graphs were generated with the ggplot2 package.^1^ A multilevel meta-analysis was conducted to compare the effect of exercise on cognitive function to that of the control group, using between-group standardized mean difference (SMD) formula: (Equation 1; 33). For studies reporting continuous outcomes, the SMD between groups was calculated as the difference in mean changes from baseline to post-intervention, divided by the pooled standard deviation of both groups:

$${\mathrm{SMD}}_{\backslash \text{between}}=\frac{\left({\overset{-}{x}}_{1,\backslash \text{post}}-{\overset{-}{x}}_{1,\backslash \mathrm{pre}})-({\overset{-}{x}}_{2,\backslash \text{post}}-{\overset{-}{x}}_{2,\backslash \mathrm{pre}}\right)}{s_{\backslash \text{pooled}}}$$ (1)

For studies that did not report the standard deviation (SD) of change scores, we estimated the variance of change scores formula: (Equation 2; 33):

Var(Δ)=Var(pre)+Var(post)−2r⋅SD(pre)⋅SD(post) (2)

where r is the pre-post correlation coefficient. We assumed a pre-post correlation coefficient of r=0.7 , a commonly used value in educational and psychological research (33). A commonly used value in educational and psychological research. We also conducted sensitivity analyses using alternative values of r=0.5 and r=0.7 to assess the robustness of our results (34). Hedges’ g correction was applied to the SMD to adjust for the small sample bias (35). The equation to convert uncorrected SMDs/Cohen’s d to Hedges’ g correction formula: (Equation 3; 83):

$$\begin{array}{c} g=\mathrm{SMD}\times (1-\frac{3}{4n-9}) \end{array}$$ (3)

Effect sizes (g) were interpreted as trivial (< 0.20), small (0.20–0.49), moderate (0.50–0.79) and large (≥ 0.80). Effect sizes from the same study are likely more similar than effect sizes from different studies (36).

Thus, the inclusion of multiple effect sizes from a single study violates the assumption of independence in effect sizes in traditional meta-analyses [e.g., (37, 38)]. As such,a three-level meta-analysis (i.e., a multilevel model) was used to account for dependencies among effect sizes from the same study (39). A multilevel meta-analysis accounts for the hierarchical nature of the data (e.g., effect sizes nested within studies) and, in so doing, the extraction of multiple effects from each study preserves information improving statistical power (36). This approach also decomposes the variance components of the pooled effect into sampling variance of the observed effect sizes (level 1), and variance within (level 2) and between studies (level 3).

As all multilevel models included moderators, statistical indices of heterogeneity were evaluated using I2 and τ2, which represented relative and absolute values of residual heterogeneity or the amount of the unaccounted for variability that is due to residual heterogeneity (40). This heterogeneity was then partitioned across two levels (i.e., within-study and between study heterogeneity).

To explore sources of heterogeneity, subgroup analyses were conducted for the following variables: exercise intensity, disease severity, exercise type, exercise frequency, total weekly exercise duration, total intervention duration, and single-session duration. Publication bias was assessed through the inspection of contour-enhanced funnel plots and Egger’s bias tests (41). Data points with absolute standardized residual values greater than 2 were identified as outliers. Then, a sensitivity analysis was conducted by re-running the meta-analysis after excluding these outliers to assess their impact on the results (42).

To confirm potential moderators of treatment effects, random effects meta-regression analyses were performed via the maximum likelihood estimation method for model building. The dose–response relationship was assessed using linear, quadratic, and cubic polynomial terms, with the optimal model selected based on the Akaike information criterion corrected (AICc) (43). Given the potential simultaneous influences of multiple factors, this study also conducted multivariate meta-regression following univariate analysis to better elucidate factors affecting treatment efficacy.

## 3 Results

**Figure 2:**
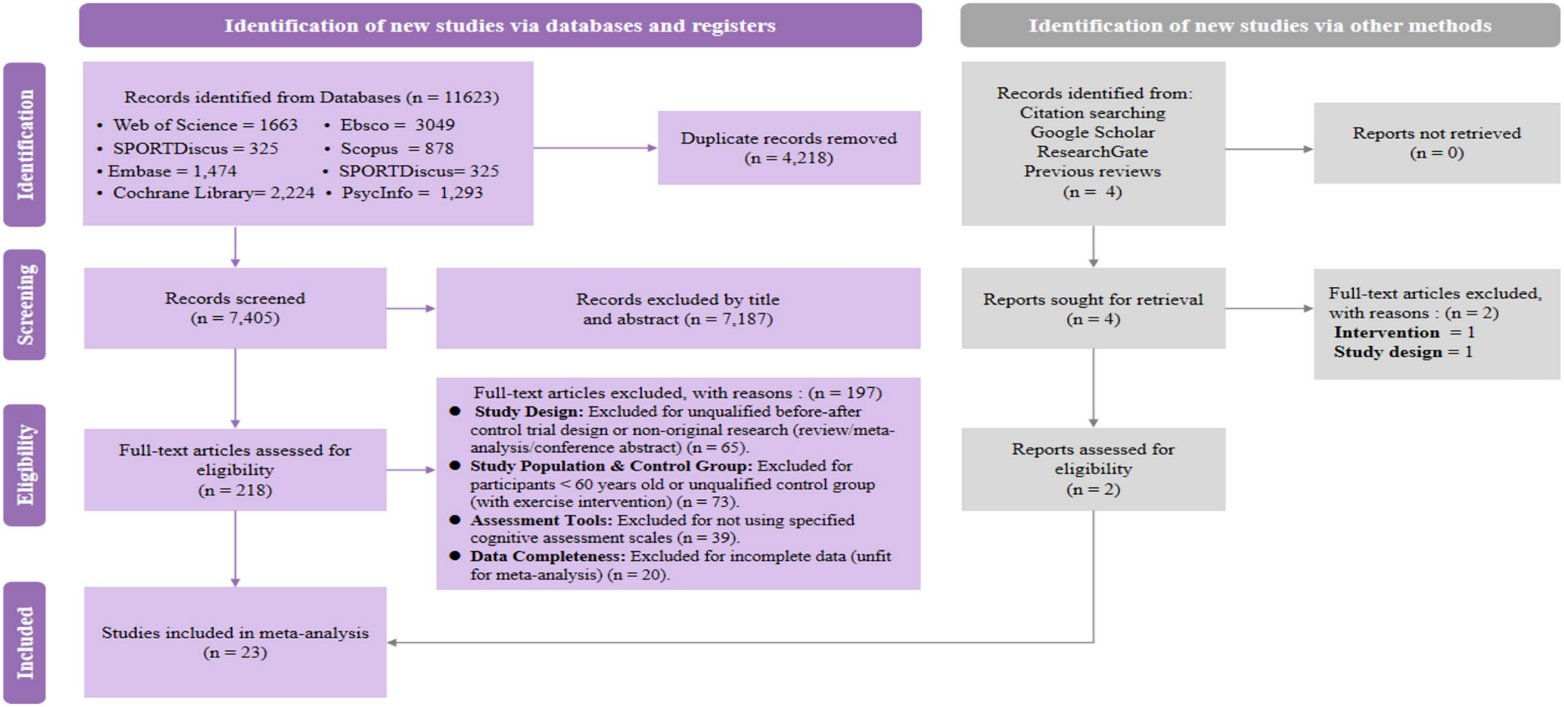
Preferred reporting items for systematic reviews and meta-analyses (PRISMA) flow diagram illustrating the various phases of the literature search and study selection process.

Initial database searches across the eight sources identified 11,623 potentially relevant records. After removing duplicates, 218 studies remained eligible for abstract screening. Following full-text review, 23 studies met the inclusion criteria and were included in the primary analysis (44–64). The literature search and selection process are summarized in Figure 2.

### 3.1 Study characteristics

This meta-analysis included a total of 1,868 participants, with 1,068 assigned to the intervention group and 780 to the control group. The gender distribution varied widely, with the proportion of female participants ranging from 30.3 to 100%. The age of participants spanned from pre-older adults (67.4 years) to advanced older adults (85.4 years). Specifically, the analysis comprised studies focusing on pre-older adults populations [*n* = 8 studies: Vreugdenhil et al. (85); Sobol et al. (60); David et al. (49); Hoffmann et al. (65); Christofoletti et al. (48); Parvin et al. (45); Morris et al. (54); Papatsimpas et al. (66)], middle-older adults populations [*n* = 8 studies: Yang et al. (87); Arcoverde et al. (46); Swinnen et al. (67); Enette et al. (51); Yu et al. (52); Shaw et al. (83); Angiolillo et al. (44); Henskens et al. (63)], advanced older adults populations [*n* = 5 studies: Eggermont et al. (50); Toots et al. (68); Kemoun et al. (56); Huang et al. (69); Van de Winckel et al. (86)], and studies in which participant ages were not specified [*n* = 2 studies: Cavalcante et al. (47); Angiolillo et al. (44)]. These studies were conducted across 10 countries (6 European, 3 Asian, and 1 African countries).

The duration of exercise interventions varied considerably, ranging from 6 to 26 weeks. Exercise regimens were typically implemented 2 to 5 times per week, with 3 sessions per week being the most common frequency (reported in 8 studies). Individual session duration ranged from 20 to 60 min, with intensity categorized as light to moderate (corresponding to 40–80% of maximum heart rate or 65–75% of relative heart rate). Exercise types included aerobic exercise (12 studies), resistance training (5 studies), balance training (1 study), combined training (3 studies), Tai Chi (1 study), dance-based games (1 study), and stretching/flexibility training (1 study). Two studies reported mild adverse events such as knee or ankle pain and delayed-onset muscle soreness (54, 65), while one study noted adverse reactions unrelated to the intervention (63). Detailed characteristics of the study participants are provided in the Tables 1–3.

**Table 1:** Participant information profile.

Study	Participants (exercise vs control)			
	MMSE score	Sample size	Age	Disease type
Arcoverde et al. (46)	20.4 ± 2.7 vs. 19.9 ± 3.4	10 vs. 10	78.5 (64–81.2) vs. 79 (74.7–82.2)	Mild Alzheimer’s disease
Swinnen et al. (67)	18 ± 4.4 vs. 17 ± 4.2	23 vs. 22	84.7 ± 5.6 vs. 85.3 ± 6.5	Major Neurocognitive Disorder (including Alzheimer’s disease, vascular dementia, mixed dementia, frontotemporal degeneration, Lewy body disease)
Cavalcante et al. (47)	< 26(not report), The inclusion criteria were based solely on the MoCA score, with no reported MMSE scores	25 VS 25	≥65	Subjective cognitive complaints
Christofoletti et al. (48)	18.7 ± 1.7 VS 12.7 ± 2.1 VS 14.6 ± 1.2	17 VS 17 VS 20	70.0 ± 1.8 VS 72.9 ± 2.3 VS 79.4 ± 2.0	Mixed dementia (institutionalized older adults)
David et al. (49)	24.09 ± 3.16 VS 25.05 ± 3.6	22 VS 19	72.1 ± 5.8 VS 68.0 ± 8.2	early-stage Alzheimer’s disease (Aβ + CSF biomarkers, CDR ≤ 1)
Eggermont et al (50)	Mean MMSE = 17.7 (SD not reported) for total sample (*N* = 97)	51 VS 46	85.4 ± (not reported) (SD not reported) for total sample	Older adults patients with dementia (mixed types, nursing home residents)
Enette et al. (51)	18 (16–21) VS 18 (17–19) VS 21 (17–23)	14 VS 17 VS 21	77.9 ± 7.6(overall sample)	mild-to-moderate Alzheimer’s disease
Yu et al. (52)	21.5 ± 3.5 vs. 22.2 ± 2.7	47 vs. 24	77.3 ± 6.3(overall)	Mild-to-moderate Alzheimer’s disease dementia (clinical diagnosis, MMSE 15–26, CDR 0.5–2)
Henskens et al. (63)	13.55 ± 5.61 vs. 13.19 ± 3.67 vs. 12.14 ± 6.43 vs. 10.23 ± 5.67	22 VS 21 vs. 22 vs. 22	86.95 ± 7.21 vs. 86.05 ± 5.86 vs. 85.14 ± 4.64 vs. 84.73 ± 4.55	Dementia (including Alzheimer’s disease, vascular dementia, mixed)
Hoffmannet al (65).	23.8 ± 3.4 vs. 24.1 ± 3.8	107 vs. 93	69.8 ± 7.4 vs. 71.3 ± 7.3	mild Alzheimer’s disease (NINCDS-ADRDA criteria, MMSE ≥20)
Huang et la (69).	20.73 ± 6.57 vs. 20.80 ± 5.16 (baseline of 80 participants)	40 vs. 40	81.9 ± 6.0(overall)	mild dementia (DSM-IV criteria, CDR < 2)
Kemoun et al. (56)	12.6 (range 7–20) vs. 12.9 (range 8–19)	20 vs. 18	81.8 ± 5.3	Alzheimer-type dementia (DSM-IV criteria, MMSE <23)
Lamb et al. (70)	21.4 ± 9.6 vs. 21.8 ± 7.7	329 vs. 165	76.9 ± 7.9 vs. 78.4 ± 7.6	mild-to-moderate dementia (DSM-IV criteria, sMMSE >10)
Morris et al. (54)	25.8 ± 3.3 vs. 25.0 ± 3.2	39 vs. 37	74.4 ± 6.7 vs. 71.4 ± 8.4	early-stage Alzheimer’s disease (probable AD, CDR 0.5 or 1)
Papatsimpas et al. (66)	74.04 ± 7.42 vs. 70.51 ± 8.83 vs. 70.53 ± 8.83	57 vs. 57 vs. 57	77.22 ± 5.73(overall)	mild Alzheimer’s disease (MMSE 20–24)
Shaw et al. (83)	18.36 ± 3.34 vs. 18.8 ± 5.77	20 vs. 20	82.21 ± 6.62 vs. 78.5 ± 6.69	mild-to-moderate Alzheimer’s disease (NINCDS-ADRDA criteria)
Hoffmannet al (65).	23.8 ± 3.4 vs. 24.1 ± 3.8	107 vs. 93	69.8 ± 7.4 vs. 71.3 ± 7.3	mild Alzheimer’s disease (NINCDS-ADRDA criteria, MMSE ≥20)
Sobol et al. (84)	25.1 ± 2.9 vs. 25.5 ± 3.3	29 vs. 26	69.2 ± 6.9 vs. 68.9 ± 7.2	mild Alzheimer’s disease (NINCDS-ADRDA criteria, MMSE ≥20)
Toots et al. (68)	15.4 ± 3.4 vs. 14.4 ± 3.5	93 vs. 93	84.4 ± 6.2 vs. 85.9 ± 7.8	Dementia (including vascular dementia, Alzheimer’s disease, mixed, other)
Vreugdenhil et al. (85)	22.9 ± 5.0 vs. 21.0 ± 6.3	20 vs. 20	73.5 ± 8.7 vs. 74.7 ± 8.8	Alzheimer’s disease (NINCDS-ADRDA criteria, DSM-IV)
Van de Winckel et al. (86)	12.87 ± 5.01 vs. 10.80 ± 5.01	15 vs. 10	81 (range67-92); Control: 81 (range 66–92); SD notreported	Alzheimer’s disease (NINCDS-ARDRA criteria) and multiple infarct dementia
Yang et al. (87)	21.33 ± 2.24 vs. 20.00 ± 3.50	25 VS 25	72.00 ± 6.69 vs. 71.92 ± 7.28	mild Alzheimer’s disease (MMSE 10–24)
Angiolilloet al (44).	20.24 ± 1.99 vs. 17.49 ± 3.89	9 vs. 13	78.89 ± 6.68 vs. 78.92 ± 8.04	mild-to-moderate Alzheimer’s disease (NIA-AA criteria, MMSE 9–24)

**Table 2:** Interference means information feature table.

Study	Interventions					
	Type	Intensity	Duration of a single exercise session (minutes)	Frequency (times/week)	Weekly exercise duration (minutes)	Length (weeks)
Arcoverde et al. (46)	Treadmill training	Low and medium	30	2	60	16
Swinnen et al. (67)	Exercise games	Mild to moderate	15	3	45	8
Cavalcante et al. (47)	RE Group VS REI Group	moderate-to-high	30	3	90	12
Christofoletti et al. (48)	Interdisciplinary program VS Physiotherapy alone	Moderate-to-vigorous	120 vs. 60	5 vs. 3	600 vs. 180	24
David et al. (49)	Multicomponent exercise (Supervised: aerobic + resistance + coordination Home-based: moderate-to-vigorous training + stretching/toning)	moderate	60 vs. 45	1 vs. 3	120	26
Eggermont et al (50)	Treadmill-free walking	moderate	30	5	150	6
Enette et al. (51)	Continuous aerobic training VS Interval aerobic training	Moderate-to-vigorous	30	2	60	9
Yu et al. (52)	Moderate-intensity cycling with adjustment period	Moderate-to-vigorous	20–50	3	150	26
Henskens et al. (63)	ADL training + multicomponent exercise vs. ADL training + social activity vs. Multicomponent exercise + care-as-usual	Moderate-to-intensive	30–45	3	90–135	26
Hoffmannet al (65).	Aerobic exercise (ergometer bicycle, cross trainer, treadmill) + initial lower extremity strength training	Moderate-to-high	60	3	180	16
Huang et la (69).	Modified Tai Chi	Low	20	3	60	40
Kemoun et al. (56)	Multicomponent training	Moderate	60	3	180	15
Lamb et al. (70)	Moderate-to-high intensity aerobic and strength exercise	Moderate-to-high	60–90	3	180–240	16
Morris et al. (54)	Supervised aerobic exercise; Non-aerobic stretching	Moderate	AEx: 30–60 min(depending on weeklyfrequency); ST: 30–60 min (matched to AEx)	3–5	150	26
Papatsimpas et al. (66)	30-min walking; 45-min resistance training; Resistance training	Moderate	30 vs. 40–45	5 vs. 3	270–285	12
Shaw et al. (83)	Multidimensional group exercise	Self-selected	45	3	135	8
Sobol et al. (84)	Aerobic exercise + initial lower extremity strength training	Moderate-to-high	60	3	180	16
Sobol et al. (60)	aerobic exercise(same as ADEX Trial: ergometer bicycle, cross trainer, treadmill)	Moderate-to-high	60	3	180	16
Toots et al. (68)	High-Intensity Functional Exercise(HIFE) program - functional weight-bearing exercises	High	45	2.5	112.5	16
Vreugdenhil et al. (85)	Home-based exercise	Moderate	30	Daily	210	16
Van de Winckel et al. (86)	Music-based group exercise	Moderate	30	Daily	210	12
Yang et al. (87)	Moderate-intensity aerobic exercise: cycling training	Moderate	40	3	120	12
Angiolilloet al (44).	Nordic Walking+ comprehensive interventions	Low and medium	60	2	120	24

**Table 3:** Interference result information feature table.

Study	Comparator	Outcomes
		CF=cognitive function, NS=neuropsychiatric symptoms, ADL (activities of daily living)
Arcoverde et al. (46)	Only maintained clinical and pharmacological treatment	Δ CAMCOG: Intervention +6.1 ± 6.7 vs. Control −6.1 ± 4.3 Δ MMSE: Intervention +0.3 ± 2.4 vs. Control −2.1 ± 0.8
Swinnen et al. (67)	Watching preferred music videos (seated, 15 min, 3×/week)	SRTT (ms): Intervention 1426.6 ± 333.1 vs. Control 5292.9 ± 4893.4 (post-intervention) MoCA: Intervention 12.1 ± 5.2 vs. Control 5.7 ± 4.0 (post-intervention)
Cavalcante et al. (47)	Health Education Control Group: Weekly health lectures	MoCA (post-intervention): RE Group 12.1 ± 5.2, REI Group 4.7 ± 4, Control 14.6 ± 1.2 MMSE (post-intervention): RE Group 18.7 ± 1.7, REI Group 20.2 ± 1.6, Control 14.8 ± 1.3
Christofoletti et al. (48)	No motor intervention; only maintained clinical/medication treatment	Δ MoCA: Significant correlation between *Δ*VO₂max and cognitive improvement in intervention group (*p* = 0.039)
David et al. (49)	Monthly psychoeducational lectures(lifestyle, disease management); no structured exercise	Memory Domain: No significant group×time interaction (*F* = 0.15, *p* = 0.86) Executive Function Domain: No significant group×time interaction (*F* = 0.37, *p* = 0.69)
Eggermont et al (50)	30-min social visits (same frequency, one-on-one conversations, no physical activity)	BCSB subtest 1: Intervention 7.8 ± 1.3 vs. Control 7.9 ± 1.5 (post-intervention) BCSB subtest 2: Intervention 3.5 ± 1.0 vs. Control 4.1 ± 1.1 (post-intervention) BCSB subtest 3: Intervention 9.2 ± 0.9 vs. Control 6.4 ± 1.1 (post-intervention) All group×time interactions non-significant (*p* > 0.05)
Enette et al. (51)	Weekly 30-min interactive information sessions(health benefits of physical activity); no exercise	Δ MMSE: No significant difference between groups (*p* > 0.05) Δ RAVLT: No significant difference between groups (*p* > 0.05) Δ Digit Span: No significant difference between groups (*p* > 0.05)
Yu et al. (52)	Low-intensity stretching (<20% HRR, RPE 9); matched frequency and session duration	Δ ADAS-Cog at 6 months: Correlation with cardiorespiratory fitness change (*r* = −0.34, *p* < 0.05) Δ ADAS-Cog at 12 months: No significant correlation
Henskens et al. (63)	SADL/SCO: Social activities(tea drinking, 3 times/week) PCO/SCO: Usual care	CF: Not reported in original study
Hoffmannet al (65).	Usual care (outpatient visits every 6 months; no exercise)	Δ SDMT: Correlation with intervention adherence (ITT: *ρ* = 0.21, *p* = 0.039; per-protocol: *ρ* = 0.20, *p* = 0.048)
Huang et la (69).	Routine treatments; no Tai Chi	MoCA: Significant group×time interaction (*F* = 5.71, *p* = 0.01) MoCA at 5 months: Tai Chi 19.47 ± 5.91 vs. Control 18.26 ± 5.64 MoCA at 10 months: Tai Chi 20.56 ± 5.23 vs. Control 17.89 ± 6.02
Kemoun et al. (56)	Nursing home activities (pottery, painting, soft gymnastics, outings); no structured exercise	ERFC (post-intervention): Intervention 30.38 vs. Control 26.81 (*p* < 0.01)
Lamb et al. (70)	Usual care (outpatient visits every 6 months，no structured exercise)	ADAS-Cog at 12 months: Intervention 25.2 ± 12.3 vs. Control 23.8 ± 10.4
Morris et al. (54)	Non-aerobic stretching/toning; same frequency/duration as AEx	Δ DAD: AEx group improved (+1.5 points) vs. ST group declined (−4.5 points) (*X*^2^ = 8.2, *p* = 0.02) Memory Composite: No significant group×time interaction (*p* = 0.66)
Papatsimpas et al. (66)	No exercise; usual care	Executive Function Composite: No significant group×time interaction (*p* = 0.27)ACE-R at 12 weeks: Group A (walking+resistance) 79.25 ± 6.46, Group B (resistance only) 75.7 ± 8.61, Group C (control) 64.28 ± 6.51 (*p* < 0.001 for A vs. C, B vs. C)
Shaw et al. (83)	No exercise; usual care	Δ MMSE: Intervention +2.0 points (*p* = 0.023) vs. Control −0.5 points (*p* > 0.05)
Sobol et al. (84)	Usual care (outpatient visits every 6 months; no structured exercise)	Δ SDMT: Correlation with dual-task performance (ITT: *ρ* = 0.21, *p* = 0.039; per-protocol: *ρ* = 0.20, *p* = 0.048)
Sobol et al. (60)	Usual care (no structured exercise)	Δ SDMT: Correlation with ΔVO₂peak (*ρ* = 0.36, *p* = 0.010)
Toots et al. (68)	Seated attention control (conversation, music, pictures)	Δ MMSE at 4 months: No significant group difference (*p* = 0.644) Δ MMSE at 7 months: No significant group difference (*p* = 0.056) Δ ADAS-Cog: No significant group difference (*p* = 0.491) Δ Verbal Fluency: No significant group difference (*p* = 0.241)
Vreugdenhil et al. (85)	Nursing home usual activities (20 min daily walking, group aerobics, occupational/art therapies)	Δ MMSE: Intervention +2.6 vs. Control −0.8 (*p* = 0.001) Δ ADAS-Cog: Intervention −7.1 vs. Control +2.3 (*p* = 0.001)
Van de Winckel et al. (86)	Daily one-to-one conversation (no music/movement)	Δ MMSE: Intervention +2.66 (12.87 → 15.53, *p* = 0.001) Control group MMSE: 10.80 → 10.10 (change not reported for significance)
Yang et al. (87)	Health education (AD-related lectures, no structured exercise)	Δ MMSE: Intervention +1.50 (21.33 → 22.83) vs. Control −0.50 (20.00 → 19.50) (*p* = 0.000 for group comparison) Δ ADAS-Cog: Intervention −2.87 (17.50 → 14.63) vs. Control +1.20 (17.80 → 19.00) (*p* = 0.004 for group comparison)
Angiolilloet al (44).	Comprehensive interventions (the same as exercise group) without NW	Δ FAB: Intervention improved vs. Control worsened (p = 0.04) Δ RVLT-D: Intervention stable vs. Control declined (p = 0.02)

Risk of bias was assessed using the ROB 2.0 tool. Based on this assessment, 13 studies were rated as having low risk of bias (i.e., high quality) (47, 50, 52, 54, 60, 63, 65, 67–70, 85). Four studies were identified as having a high risk of bias (45, 48, 83, 86). Detailed results of the methodological quality assessment, including scoring, are provided in Supplementary Table S3.

### 3.2 Primary analysis

Overall, exercise had a significant positive effect on improving cognitive function in patients with AD (*g* = 0.22, 95% CI: 0.02–0.41, *p* < 0.05). A high degree of heterogeneity was observed across studies [Q (df = 142) = 1318.1, *p* < 0.001; *I*^2^ = 73%]. Between-study and within-study variability due to sampling error were both minimal (*τ*^2^ < 0.01, *σ*^2^ = 0.18). Subgroup analyses are presented in Table 1. For the detailed GRADE evaluation process of each subgroup, please refer to Appendix 3 (Table 4).

**Table 4:** Subgroup analysis examining the effects of disease severity, exercise type, exercise intensity, single exercise duration, frequency and weekly exercise duration on treatment of Alzheimer’s disease.

Subgroup	Individual estimates		Between-condition comparison			
	*N*	g (95% CI)	*p* value	Difference g (95% CI)	*p* value	Grade
Age(year)						
≤75 (reference group)	46	0.46 (0.18,0.73)	<0.01	Reference		Low
>75	97	0.10 (−0.14,0.34)	0.42	−0.36 (−0.61,-0.11)	0.0053	Very Low
Frequency(times/week)						
≤3 (reference group)	54	0.27 (−0.06,0.60)	0.10	Reference		Very Low
3–5	60	0.06 (−0.19,0.31)	0.62	0.08 (−0.40，0.56)	0.32	Moderate
>5	29	0.54 (0.12,0.96)	<0.05	0.27 (−0.26，0.81)	0.31	Very Low
Single exercise duration(min)						
≤30 (reference group)	57	0.05 (−0.32,0.42)	0.80	Reference		Very Low
30–60	45	0.12 (−0.19, 0.43)	0.43	0.08 (−0.40,0.56)	0.13	Low
≥60	41	0.47 (0.13,0.80)	0.0066	0.42 (−0.08,0.92)	0.0968	Moderate
Total interference duration(year)						
≤ 1 (reference group)	80	0.36 (0.08,0.64)	<0.05	Reference		High
1–2	30	0.09 (−0.19,0.37)	0.53	−0.27 (−0.68,0.13)	0.18	Low
≥ 2	33	0.09 (−0.21,0.40)	0.54	−0.27 (−0.68,0.15)	0.21	Very low
Weekly exercise duration(min)						
<90 (reference group)	60	0.10 (−0.25,0.44)	0.58	Reference		Moderate
≥ 90	83	0.27 (0.04,0.51)	< 0.05	0.18 (−0.24,0.60)	0.41	Low

Following sensitivity analysis, the pooled effect size of exercise intervention on cognitive function in AD patients was g = 0.19 (95% CI: 0.05–0.33), which falls within the small effect size range (71). To facilitate clinical interpretation, this effect size corresponds to an approximate mean improvement of 0.5–1.0 points on the Mini-Mental State Examination (MMSE), based on pooled standard deviations from previous studies (71). In the context of Alzheimer’s disease—a progressive neurodegenerative condition—even small magnitudes of cognitive maintenance or slowed decline can hold clinical relevance (72). Compared with previous meta-analyses of exercise interventions in AD populations, our effect size is highly consistent with those reported by Groot et al. (73) (SMD = 0.27) and the observational findings of Öhman et al. (74), suggesting that exercise confers a stable but limited cognitive benefit in AD patients.

**Figure 3:**
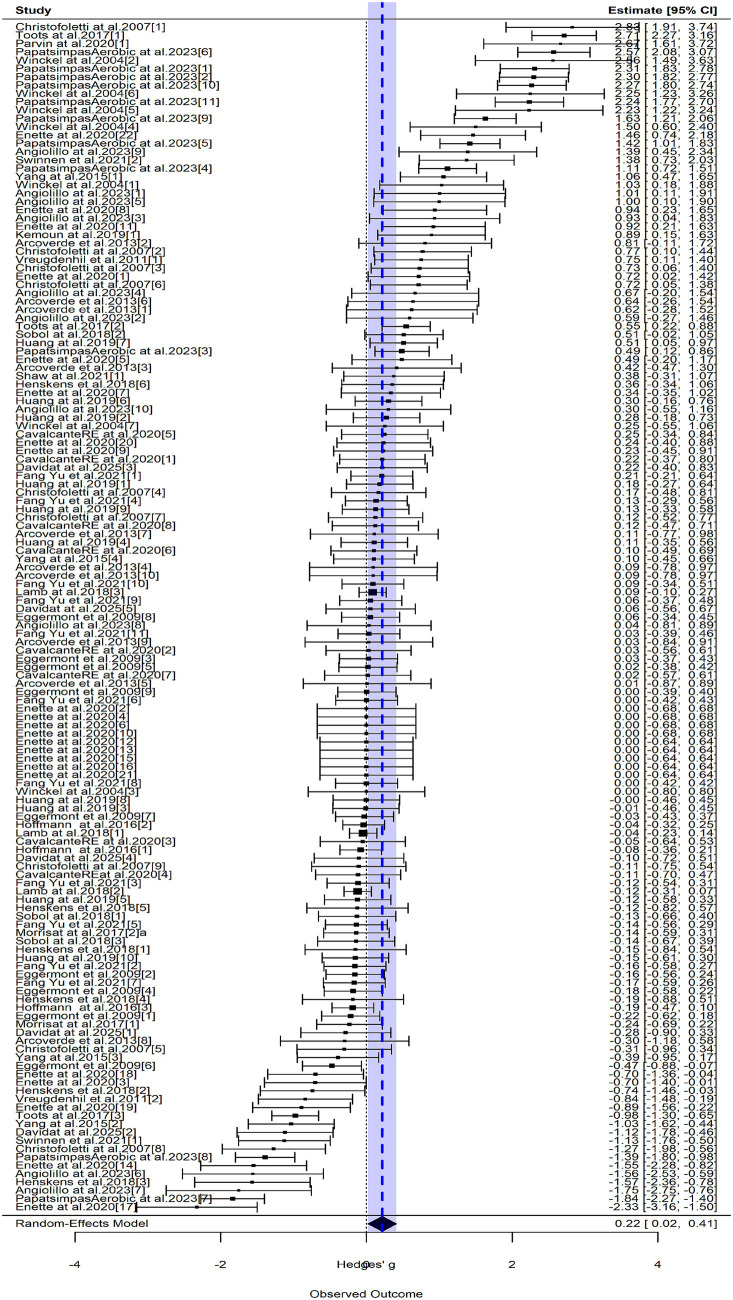
forest plot of the whole study. Some studies appear multiple times in this forest plot because multiple effect sizes were extracted from the same study (e.g., separate cognitive outcomes, multiple intervention arms, or different follow-up time points). This is consistent with the multilevel meta-analytic approach used in this study, which accounts for the nesting of multiple effect sizes within studies. A total of 23 studies contributed 143 effect sizes to the analysis. Squares represent individual effect sizes, with square size proportional to study weight. Horizontal lines indicate 95% confidence intervals. The diamond at the bottom represents the pooled effect size. A high-resolution version of this forest plot is available in the Supplementary materials for detailed viewing.

Funnel plot inspection (see Figure 3) and Egger’s test indicated potential publication bias (intercept = 0.87, *p* = 0.01). Analysis of standardized residuals identified 27 outliers. At the same time, the cross-validation method of Cook’s distance and one leave method is used to verify the stability of the results. The results show that the model tends to be stable when 27 studies are deleted. After their exclusion, exercise showed only a trivial effect on cognitive improvement (*g* = 0.19, 95% CI: 0.05–0.33, *p* < 0.001). Heterogeneity was also reduced [Q (df = 115) = 219.98, *p* < 0.0001; *I*^2^ = 55%], and sampling error-related variability remained negligible between studies (*τ*^2^ < 0.01) and within studies (*σ*^2^ = 0.08).

### 3.3 Subgroup analysis

#### 3.3.1 Age

Among the 23 included studies, 21 explicitly confirmed a baseline diagnosis of AD. All participants were older adults, covering various age stages of AD, which provided a sufficient sample to explore the moderating effect of age on exercise intervention outcomes. The analysis indicated that age was not a key moderator of cognitive improvement through exercise in AD patients. To further examine this relationship, a linear model was fitted. The results showed an effect size g of 0.35 (95% CI: −0.70 to 1.41, *p* > 0.05), illustrating no significant linear association between age and intervention efficacy. Heterogeneity across studies was moderate (*I*^2^ = 49.14%), and was not primarily explained by age. Additionally, to assess potential nonlinearity, a restricted cubic spline model with three knots was applied. This analysis also showed no significant nonlinear association (*p* > 0.05), with heterogeneity (*I*^2^ = 48.91%) remaining consistent with the linear model. Together, these findings confirm that age does not significantly moderate the effect of exercise on cognitive function in AD patients.

Based on meta-regression analysis, age did not show a significant moderating effect on exercise outcomes (*p* > 0.05) and explained none of the between-study heterogeneity (*R*^2^ = 0.00%). This finding is consistent with previous meta-analyses (73), suggesting that age is not a robust moderator of cognitive benefits from exercise in AD patients.

#### 3.3.2 Frequency

To examine the influence of exercise frequency on cognitive improvement in AD patients, we systematically analyzed the 23 included studies focusing on older adults AD populations. The results indicated that exercise frequency significantly moderated cognitive enhancement (*p* < 0.001), suggesting that the effect of exercise intervention on cognitive function differed substantially across frequency levels. To accurately characterize the relationship between frequency and efficacy, multiple fitting models were compared. A cubic model with three knots best described the association between exercise frequency and cognitive improvement. Residual heterogeneity was significant [QE (df = 113) = 192.54, *p* < 0.001], and the spline terms as moderators were also statistically significant [QM (df = 2) = 13.9]. Further analysis showed that the cognitive benefit of exercise was most pronounced when the weekly frequency exceeded five sessions (Figure 3).

Exercise frequency demonstrated a significant moderating effect on cognitive improvement (*p* < 0.001), with cubic spline modeling indicating notably enhanced effect sizes when frequency exceeded five sessions per week. This finding provides preliminary evidence for a dose–response relationship, but given the limitations of univariate analysis, confirmation through prospective trials is warranted (75).

#### 3.3.3 Total intervention duration

Using a subgroup analysis approach, this study systematically examined the moderating effect of total intervention duration on the efficacy of exercise for improving behavioral and cognitive function in AD patients. The overall analysis showed that total intervention duration did not have a statistically significant moderating effect on intervention efficacy [QE (df = 114) = 219.9311, *p* < 0.001], indicating that, at an overall level, exercise regimens of different durations did not produce significantly different cognitive improvements. To further investigate potential nonlinear relationships, a restricted cubic spline model with five knots was applied. The results revealed phase-specific moderation by duration: when the intervention lasted less than 7.5 weeks, there was a significant association between duration and cognitive improvement (*p* < 0.05), suggesting that extending the intervention in the initial phase could enhance its efficacy (*g* = −2.1105, *p* < 0.0001, 95% CI: −3.02 to −0.23).

However, as duration increased further, the positive effect on cognitive improvement gradually attenuated. When the total duration exceeded 7.5 weeks (*g* = −3.1196, *p* > 0.05, 95% CI: −7.05 to −0.81), additional time no longer provided significant added benefit, suggesting that exercise intervention has a short-term effective window.

Total intervention duration showed a phase-specific association with cognitive improvement: significant effects were observed during the initial phase (<7.5 weeks), followed by a tendency toward plateau. This pattern should be interpreted as an exploratory observation, potentially reflecting heightened neuroplastic responsiveness in early intervention stages (73), rather than evidence of a fixed “optimal window”.

#### 3.3.4 Single-session exercise duration

To determine whether single-session exercise duration is a key factor influencing the efficacy of exercise interventions for cognitive function in AD patients, we analyzed the 23 included exercise studies in older adults AD populations. The results indicated that single-session duration did not significantly moderate intervention outcomes (*g* = 0.03, *p* > 0.05, 95% CI: −0.11 to 0.16). Namely that, variations in single-session duration did not lead to notable differences in cognitive improvement.

To ensure the robustness of the findings, several fitting methods were used for cross-validation, including a linear model and restricted cubic spline models with different knot specifications. Results from these multiple approaches consistently showed no significant linear or nonlinear relationship between single-session exercise duration and cognitive improvement in AD patients. This suggests that single-session duration is not an independent key parameter determining the outcome of exercise interventions.

Single-session duration did not show a significant moderating effect on cognitive improvement (*p* > 0.05), and none of the fitted models reached statistical significance. This suggests that session duration is not an independent key moderator; however, given the heterogeneity in reporting across original studies, this conclusion requires further validation.

#### 3.3.5 Total weekly exercise duration

In contrast to the findings for single-session duration, subgroup analysis clearly showed that weekly exercise duration was a key moderator of exercise efficacy in improving cognitive function in AD patients. To further examine the relationship between weekly duration and intervention efficacy, as well as its role in explaining between-study heterogeneity, a cubic polynomial model was fitted (*g* = 0.37, *p* < 0.05, 95% CI: 0.05 to 0.68).

The results revealed a significant nonlinear association between weekly exercise duration and cognitive improvement in AD patients. This moderator explained 17.20% of the between-study heterogeneity, indicating that differences in weekly exercise duration partly accounted for the variation in intervention effects across studies. Including weekly duration as a moderator effectively reduced the degree of between-study heterogeneity.

Total weekly exercise duration demonstrated a significant moderating effect on cognitive improvement (*p* < 0.05), explaining 17.20% of between-study heterogeneity. This finding provides preliminary evidence for a dose–response relationship with total exercise volume, although the nonlinear model suggests potential threshold effects (18, 19).

### 3.4 Multivariate analysis

**Figure 4:**
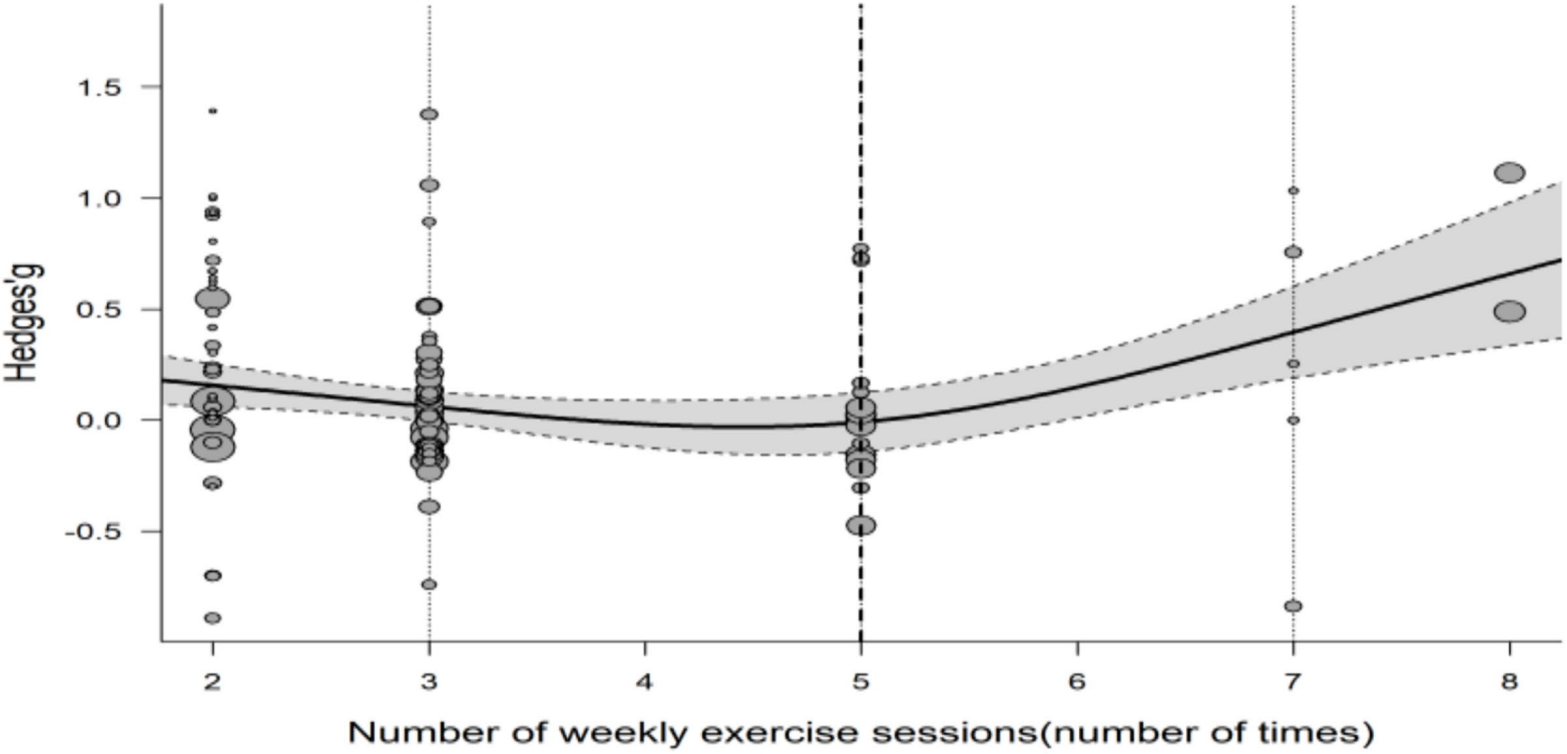
Cubic spline regression model depicting the relationship between exercise frequency (expressed as sessions per week) and cognitive function improvement in patients. Thin dashed vertical lines indicate the positions corresponding to 3, 5, and 7 sessions per week; thick dashed vertical lines represent the critical points prior to the rapid improvement of cognitive function. The shaded area denotes the 95% confidence interval.

Meta-regression analysis indicated that several factors influencing exercise-induced cognitive improvement in AD patients showed significant dose–response relationships with certain parameters (Tables 1, 5; Figure 4).

**Table 5:** Effects of exercise on cognitive function in patients with Alzheimer’s disease: meta-regression coefficients of linear, quadratic, and cubic models based on AICC values.

Variable	b₀*	95%CI^†^	b₁^‡^	95%CI^§^	p^‖^	AICc^¶^	AICc₂**	AICc₃^††^
Age								
	0.35	−0.71, 1.41	−0.003	−0.02, 0.01	0.65	52.83	57.70	51.00
	0.12	−0.76, 1.00	−0.06	−0.12, 0.00	0.03	54.41	55.32	46.42
	0.27	−0.6, 1.13	−0.06	−0.10, 0.00	0.03	52.83	57.70	51.00
Frequency								
	0.03	−0.14, 0.20	0.02	−0.02, 0.07	0.33	55.31	55.40	54.15
	0.35	0.11, 0.59	−0.10	−0.18, −0.01	0.02	50.32	60.98	66.11
	0.94	−0.58, 2.46	−0.09	−0.17, −0.01	0.02	59.31	60.98	66.11
Single-session exercise duration								
	0.03	−0.11, 0.16	0.002	−0.001, 0.005	0.16	59.37	62.11	42.49
	−0.10	−0.42, 0.22	0.001	−0.01, 0.01	0.88	59.37	62.11	42.49
	−0.24	−1.58, 1.10	−0.001	−0.01, 0.01	0.98	59.31	60.98	66.11
Total weekly exercise duration								
	0.06	−0.04, 0.17	0.0003	−0.0002, 0.0009	0.25	59.37	62.11	42.49
	−1.05	−1.90, −0.20	−0.11	−0.20, −0.02	0.02	54.33	62.40	46.45
	0.37	0.05, 0.68	−0.006	−0.01, 0.00	0.06	59.37	62.11	42.49
Total intervention duration								
	0.11	−0.02, 0.25	−0.0001	−0.01, 0.01	0.98	55.31	55.40	54.15
	−0.25	−0.72, 0.22	0.04	−0.01, 0.09	0.15	50.32	60.98	66.11
	−0.79	−1.62, 0.04	−2.11	−3.02, −0.23	0.0001	59.31	60.98	66.11

^†^95% CI for pooled mean difference; ^‡^*p* value for pooled mean difference; ^§^I2 measure of heterogeneity between studies expressed as percentage; ¶*p* value from the test of heterogeneity; **p value for funnel plot asymmetry; ^††^*p* value for the test of moderation effect.

To examine the combined influence of multiple factors on cognitive improvement, a model-based inference approach was used to identify core predictors and inform the optimization of exercise regimens for AD patients. Correlation analysis revealed a strong positive correlation between exercise frequency and weekly exercise duration (r > 0.5), suggesting collinearity (Table 2). To avoid bias, weekly exercise duration was excluded from subsequent modeling (Table 6).

**Table 6:** Pearson correlation coefficients of key exercise intervention variables.

Variable	Age	Single session exercise duration	Frequency	Total weekly exercise duration	Total intervention duration
Age	1.00	−0.31	0.09	−0.29	0.03
Single session exercise duration	−0.31	1.00	0.28	0.18	0.47
Frequency	0.09	0.28	1.00	0.60	0.06
Total weekly exercise duration	−0.30	0.18	0.60	1.00	0.42
Total intervention duration	0.03	0.47	0.06	0.42	1.00

This table presents the Pearson correlation coefficients between age, single session exercise duration, exercise frequency, total weekly exercise duration, and total intervention duration. A larger absolute value of the correlation coefficient indicates a stronger linear relationship between the variables.

**Figure 5:**
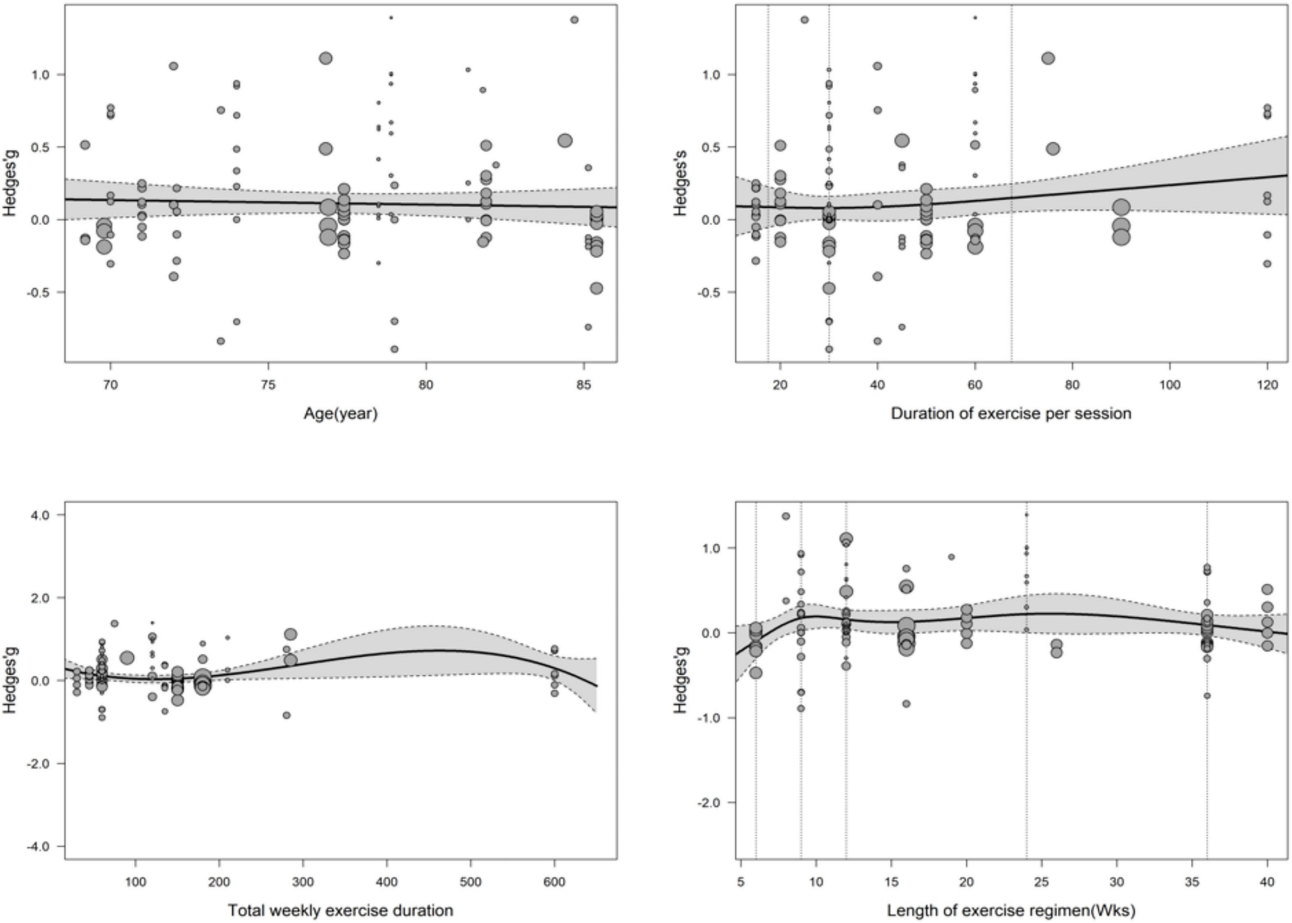
The effect of exercise on cognitive function in Alzheimer’s disease patients: results of meta-regression analysis based on multiple predictors.

**Figure 6:**
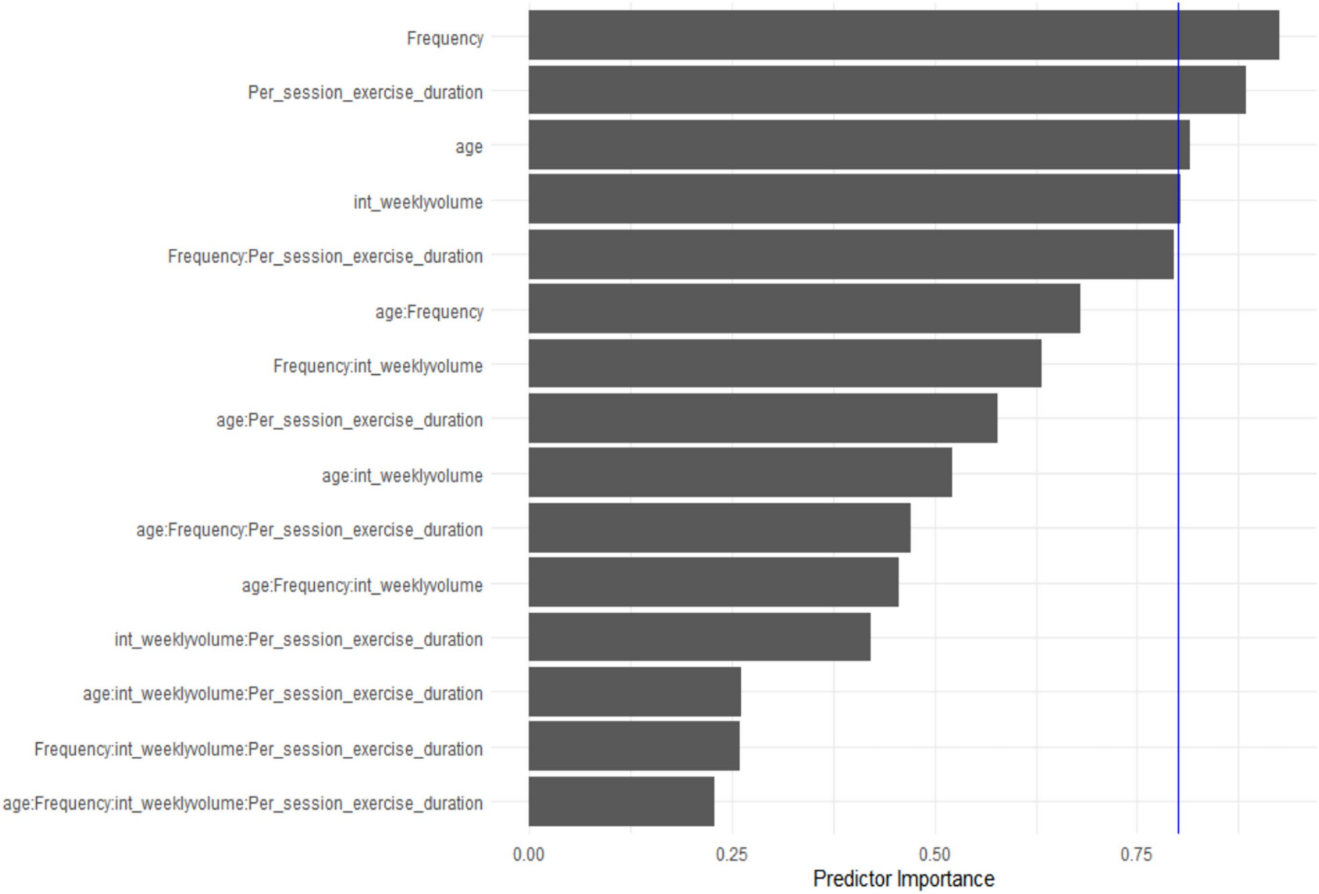
Importance ranking of influencing factors: age, exercise frequency, single-session exercise duration, total intervention duration, and their interaction effects.

Model results (see Figure 5) indicated that exercise frequency was a key predictor of cognitive improvement efficacy (importance score = 0.93). However, multiple fitting analyses across subgroups produced non-significant equations (*p* > 0.05) with low goodness-of-fit, yielding no consistent conclusions. This suggests that exercise-related cognitive benefits in AD patients are also influenced by other factors, such as individual baseline health status and disease duration (Figure 6).

## 4 Analysis and discussion

### 4.1 Key findings

This systematic review and meta-analysis demonstrated that exercise intervention significantly improves cognitive function in patients with AD (*g* = 0.22, *p* < 0.001). Subgroup analyses and meta-regression further revealed that this effect is moderated by multiple factors, with varying levels of evidence strength. Specifically, exercise frequency (*p* < 0.001) and total weekly exercise duration (*p* < 0.05) showed significant moderating effects and partially explained between-study heterogeneity, providing preliminary evidence for dose–response relationships. Total intervention duration exhibited a phase-specific association with cognitive improvement, but this should be interpreted as an exploratory observation. Age and single-session duration did not show significant moderating effects, suggesting they are not robust predictors of cognitive benefits. None of the multivariate models reached statistical significance, indicating that the current data do not support precise exercise prescription recommendations based on multiple parameters.

### 4.2 Age

Age was hypothesized to contribute to the high heterogeneity observed in this study, since all participants were older adults AD patients aged over 60 years. Previous literature has often examined the influence of age on cognitive plasticity and exercise efficacy. Some studies suggest that advanced age may reduce neuroplasticity (76), thereby diminishing cognitive benefits from exercise. For example, Huang et al. (77) reported that older adults with mild dementia showed smaller cognitive improvements. Conversely, other studies argue that age does not significantly moderate exercise outcomes in AD patients. For instance, Lamb et al. (57) noted that after controlling for disease severity and exercise dose, age did not significantly affect overall cognitive improvement. To further examine the role of age in heterogeneity, we conducted meta-regression analysis. Despite a wide age range (67.4–85.4 years), cubic spline fitting indicated that age was not a major source of heterogeneity in this meta-analysis [QE (df = 113) = 218.4475, *p* < 0.001, *R*^2^ = 0.00%]. This finding aligns with previous reports: Groot et al. (73) observed that age did not have a sustained moderate effects in dementia populations, perhaps because disease-related cognitive decline is the dominant factor. Similarly, Sobol et al. (60) and Yu et al. (52) found no obvious differs in cognitive benefits across age subgroups of AD patients.

In summary, although age may theoretically affect neural response and exercise adaptability, it was not a key contributor to heterogeneity in exercise intervention effects among the AD patients studied. It is suggested that, in patients with established AD, cognitive response to exercise is more strongly influenced by factors such as disease stage and exercise prescription characteristics than by age alone. Future research could integrate neuroimaging or biomarkers to further explore potential nonlinear relationships between age and brain plasticity across different disease stages.

### 4.3 Exercise frequency

A growing body of evidence supports this dose–response relationship. For example, Liu-Ambrose et al. (75) found that twice-weekly resistance training improved executive function in older women with mild cognitive impairment and suggested that higher frequencies may lead to greater benefits. Similarly, a systematic review by Öhman et al. (74) indicated that, among physically capable individuals, aerobic or combined exercise performed at least three times per week was associated with greater cognitive improvement in dementia patients. Indirect evidence from some of our included studies further supports the importance of frequency. For instance, Papatsimpas et al. (66) implemented a high-frequency regimen of eight sessions per week and reported a relatively large effect size. In contrast, studies using lower frequencies (e.g., 2–3 sessions per week) generally showed smaller or more variable effect sizes. Notably, although Toots et al. (68) used a frequency of only two sessions per week, they still observed significant effects through a high-intensity protocol, suggesting a potential interaction between frequency and intensity. Nevertheless, under comparable intensity conditions, increasing frequency remains an important strategy for enhancing intervention efficacy.

The present study identified a nonlinear association between exercise frequency and cognitive improvement using cubic spline modeling, with effect sizes notably increasing when frequency exceeded five sessions per week. This trend should be understood within a dose–response framework, suggesting that higher frequency may confer cumulative benefits, but it should not be interpreted as a definitive therapeutic threshold. Future studies are needed to disentangle the interactive effects of frequency, intensity, and type of exercise to clarify their independent and combined contributions in AD populations (19, 75).

### 4.4 Total intervention duration

Building on previous research that has largely focused on short-term interventions (≤ 12 weeks), this study further examined the effects of longer exercise durations (up to 36 weeks) on cognitive function in AD patients. Integrating restricted cubic spline modeling with existing evidence, this study suggests a time-sensitive association between exercise intervention and cognitive improvement in AD patients, with more pronounced effects observed during the initial phase (e.g., within the first 12 weeks) and a tendency toward plateau thereafter. This pattern should be interpreted as an exploratory observation, potentially reflecting heightened neuroplastic responsiveness in the early stages of intervention (73), rather than evidence of a fixed “optimal window.” Future research should therefore focus on how to dynamically adapt exercise programs over time to sustain neuroprotective effects. This finding aligns with existing consensus and multiple previous studies. For example, Brasure et al. (72) noted that physical activity provides small-to-moderate cognitive benefits in dementia patients in the short term (typically within 6 months), while evidence for long-term effects (> 6 months) is limited and inconsistent, and some benefits may be difficult to sustain—suggesting a “benefit plateau.” A similar pattern was observed across the 23 studies included in this meta-analysis.

Many short-term intervention studies (≤ 12 weeks) have reported significant positive effects. For instance, Swinnen et al. (78), Arcoverde et al. (46), and Yang et al. (79) all demonstrated exercise-related improvements in overall or domain-specific cognition. In contrast, with longer intervention periods, the sustainability and magnitude of improvement became more variable. Hoffmann et al. (54) found only a small effect on executive function in mild AD. Although a large trial by Lamb et al. (70) confirmed the benefits of exercise, the effect size increase was limited. Longer-term studies, such as those by Fang Yu et al. (52) and Christofoletti et al. (48), also did not show continuously increasing effects across all cognitive domains over time.

This time-benefit pattern may reflect several mechanisms. First, patients may exhibit neural adaptations to novel exercise stimuli early on, leading to relatively pronounced short-term gains. Second, as AD is a progressive neurodegenerative disorder, its underlying pathology may gradually counteract exercise-induced neuroplasticity, flattening the long-term benefit curve. Third, maintaining adherence, preventing declines in training adaptability, and dynamically adjusting programs over extended periods are challenging; fixed regimens may not consistently provide optimal stimuli.

In summary, restricted cubic spline modeling, together with existing evidence, suggests a possible time-limited window for exercise-related cognitive improvement in AD patients. Short-term interventions produce clear benefits, whereas additional gains from prolonged interventions appear limited. Therefore, in clinical practice and future research, efforts should focus on optimizing long-term programs (e.g., through periodic adjustments and multimodal combinations), enhancing adherence, and dynamically assessing neuroprotective effects with biomarkers to maximize sustained benefits from exercise.

### 4.5 Single-session exercise duration vs. total weekly exercise duration

As key dose parameters in exercise prescriptions, single-session duration and total weekly duration may influence cognitive outcomes in AD patients. Existing literature generally supports a dose–response relationship between exercise volume and cognitive benefits. For example, Northey et al. (19) reported a positive association between total weekly exercise duration and cognitive improvement, and Suzuki et al. (80) found that longer single-session durations (e.g., ≥ 30 min) were linked to better executive function and memory.

However, in the present meta-analysis of AD patients, the dose–response relationship appears more complex. We observed a significant association between total weekly exercise duration and cognitive improvement, which was well described by a cubic polynomial model [QE (df = 112) = 201.5974, *p* < 0.001]. This suggests that total weekly duration explains part of the between-study heterogeneity, partially aligning with Northey et al. (19), but indicating that the relationship may not be strictly linear.

In contrast, no stable or well-fitting model emerged for single-session duration in relation to cognitive effect sizes, implying that it is not a major independent explanatory factor for the observed heterogeneity. Potential reasons include: (1) single-session duration is often confounded by exercise type and intensity, complicating the isolation of its independent effect; (2) AD patients show considerable interindividual variation in tolerance and adherence to session length; (3) the present analysis primarily reflects long-term intervention effects, where acute responses to single sessions may be diluted in aggregated data.

These findings suggest that, for AD patients, ensuring an adequate total weekly exercise volume may be more crucial than focusing solely on single-session duration. That said, session length should still be individualized according to the patient’s condition, exercise type, and intensity, in order to achieve an effective cumulative weekly dose.

### 4.6 Multivariate analysis

Given that the effect of exercise on cognitive function in AD patients is influenced by multiple complex factors—such as exercise frequency, total intervention duration, and total weekly exercise duration (18), and because the interactions and relative contributions of these factors remain unclear, this study employed a model-based inference approach (32) to identify core predictors and optimize predictive models. Using different predictor combinations, we constructed multiple multivariate models, systematically compared their goodness-of-fit, and applied factor importance ranking algorithms to comprehensively assess the contribution of each predictor to intervention efficacy.

Notably, none of the multivariate meta-regression models we constructed reached conventional levels of statistical significance (*p* > 0.05), and all exhibited low goodness-of-fit. This finding carries important methodological and clinical implications. First, it indicates that although we identified core moderators such as exercise frequency and single-session duration, the current models have limited predictive capacity and cannot provide precise exercise prescription thresholds or combination recommendations for clinical practice. Second, the non-significant models suggest that unmeasured or underreported factors—such as disease severity, baseline cognitive status, genetic background (e.g., APOE ε4 carrier status), medication use, and social support—may play important roles in moderating the cognitive effects of exercise, beyond the variables included in our analyses (18, 19). Third, this limitation reflects a broader challenge in the field of exercise intervention research in AD: the heterogeneity across studies in the definition and reporting of exercise prescription parameters and the selection of cognitive outcome measures constrains our ability to construct robust predictive models through meta-regression (74).

Therefore, the results of our multivariate analyses should be interpreted as exploratory and hypothesis-generating, rather than as definitive conclusions that can directly inform clinical prescriptions (32). These findings provide important directions for future research: first, well-designed prospective randomized controlled trials that directly compare different exercise prescription parameters are needed to validate the dose–response associations suggested by this meta-analysis; second, individual participant data (IPD) meta-analyses should be conducted to more precisely quantify the independent and interactive effects of various moderators (18, 19); third, standardized reporting of exercise interventions using consensus statements such as the CERT (Consensus on Exercise Reporting Template) should be promoted to enhance comparability and data integration across future studies. In clinical practice, the findings of this study should serve as one of several reference points for developing individualized exercise programs, rather than as fixed prescriptions, and should be integrated with comprehensive assessment of each patient’s specific condition.

### 4.7 Effects of other potential factors

Beyond the primary factors already discussed, exercise-related cognitive improvement in AD patients may also be moderated by other variables, including exercise intensity, exercise type, and disease subtype. Although these were not included as main predictors in our multivariate modeling, their potential relevance should not be overlooked, as they are reflected to varying degrees in the included literature.

#### 4.7.1 Exercise intensity

Exercise intensity is a core element of exercise prescription. However, due to considerable variation in how intensity is defined, reported, and quantified across studies (e.g., %HRmax, %VO₂max, RPE, %1-RM), and the absence of consistent standards, we did not analyze intensity as a continuous variable in dose–response modeling.

Nonetheless, the importance of intensity is widely acknowledged. The included studies reported positive effects across different intensity levels. For example, Swinnen et al. (61) observed cognitive benefits with low-intensity exergames, while Arcoverde et al. (46) reported improvements following moderate-intensity training (60% VO₂max). Some studies indirectly compared intensity effects: Enette et al. noted that a higher-intensity group (80% HRmax) tended to show larger effect sizes in measures such as working memory, and Toots et al. (68) reported a large effect size using high-intensity functional exercise (HIFE). This suggests that, within safe limits, higher intensity may offer greater potential for cognitive gain.

Future research should employ standardized definitions and reporting of exercise intensity, and systematically examine the dose–response relationship and safety thresholds for intensity in AD cognitive intervention through RCTs directly comparing intensities or via individual participant data (IPD) meta-analyses.

#### 4.7.2 Exercise type

Exercise type represents another key dimension of exercise intervention. The included studies encompassed a variety of modalities, primarily aerobic exercise, resistance training, mixed training, mind–body exercises (e.g., Tai Chi), and game-based or functionally oriented comprehensive training. Different exercise types may affect cognitive function through distinct physiological and psychological pathways.

Evidence from the reviewed studies suggests that multiple exercise types can benefit cognitive function in AD patients. Aerobic training (65), resistance training (47), and mixed training have each shown positive effects in specific cognitive domains. Mind–body practices such as Tai Chi have also been associated with cognitive improvement in older adults with mild dementia (69) implying that the attentional control, motor learning, and mind–body coordination they involve may confer unique advantages. Furthermore, task-oriented or integrated functional training appears promising due to its relevance to daily activities and inherent cognitive engagement.

Current evidence regarding the “optimal” exercise type remains inconclusive. As noted by Öhman et al., the cognitive effects of different exercise modalities in dementia patients may depend on the cognitive domain assessed. Future studies should not only compare the relative efficacy of different exercise types but also investigate personalized type recommendations for different AD stages and clinical profiles, as well as potential synergistic effects of multimodal exercise combinations.

#### 4.7.3 Disease subtype

This study primarily included patients with AD, encompassing those diagnosed with typical AD, mild cognitive impairment (MCI) likely due to AD, and some individuals with a broad diagnosis of “dementia” or “major neurocognitive disorder” (MNCD), most of whom had AD as the primary etiology. As AD represents a continuous spectrum, disease severity may moderate the effects of exercise intervention.

Some evidence suggests that interventions delivered at earlier disease stages (e.g., MCI or mild AD) may offer greater potential for cognitive improvement. For instance, Papatsimpas et al. (66) reported a large effect size in a study focused on mild AD patients. In contrast, effect size appeared more variable in patients with moderate to severe disease, such as the MNCD population examined by Swinnen et al. (67). However, a large-scale trial by Lamb et al. (70), which included participants with mild to moderate AD, shown that exercise still conferred statistically significant overall cognitive benefits, albeit with a moderate effect size. This indicates that exercise remains beneficial even after disease onset, though its primary aims may shift toward maintaining function or slowing decline.

Moreover, AD often coexists with vascular pathology (mixed dementia) (81). Given that exercise can improve vascular risk factors, the mechanisms and extent of benefit may differ in patients with vascular comorbidities (82). Due to limited stratified reporting on AD subtypes or vascular comorbidities in the original studies, we could not conduct detailed analyses at this level. Future research should classify patients based on disease stage (80), pathological biomarkers (e.g., Aβ, tau), and comorbidity profiles, to identify subgroups that may benefit most from specific exercise interventions and advance toward precision rehabilitation.

### 4.8 Study limitations and future perspectives

Several limitations of this study should be considered when interpreting the results. First, although we aimed to examine dose–response relationships between exercise and cognition, inconsistent reporting of key parameters (e.g., exercise intensity and type) in the original studies limited precise quantification and direct comparisons. Second, while all included participants had a primary diagnosis of AD, they represented a range of disease stages (from MCI to moderate–severe dementia), which may have influenced the overall effect estimate. We were unable to conduct subgroup analyses stratified by disease severity due to insufficient data. Third, the multivariate models we developed did not reach statistical significance, suggesting that important predictors (e.g., genetic background, medication use, cognitive reserve) may have been omitted, or that model performance was limited by sample size or measurement sensitivity.

The pooled analysis revealed substantial heterogeneity across included studies (*I*^2^ = 73%) and significant publication bias as indicated by Egger’s test (*p* = 0.01), both of which limit confidence in the precision of our pooled effect size estimates. The high heterogeneity likely stems from wide variations in exercise prescription parameters (e.g., type, frequency, intensity, session duration), participant characteristics (e.g., disease severity, baseline cognitive status), and cognitive outcome measures across studies (73). Although we employed multilevel meta-analysis, subgroup analyses, and meta-regression to explore sources of heterogeneity—identifying moderators such as exercise frequency and total intervention duration—a considerable proportion of residual heterogeneity remained unexplained (residual heterogeneity QE test *p* < 0.001). This suggests that unmeasured or underreported study-level factors—such as participant adherence, social support, and genetic background—may also influence intervention effects (18, 19).

The presence of publication bias raises the possibility that studies with smaller effect sizes or null findings may be underrepresented in the literature, potentially leading to overestimation of the true effect (41). In accordance with the GRADE framework, we therefore downgraded the certainty of evidence from “high” to “moderate” to reflect these methodological limitations and their implications for the robustness of our conclusions (29). Future research should promote prospective trial registration and full publication of all results (regardless of direction or significance) to mitigate publication bias. In addition, standardized reporting of exercise intervention details using consensus statements such as the CERT (Consensus on Exercise Reporting Template) will facilitate more precise quantification of heterogeneity sources and enable individual participant data (IPD) meta-analyses (32).

Given these limitations, future research could advance in several directions. First, standardized reporting of exercise parameters (e.g., intensity, type) should be encouraged to facilitate more accurate dose–response modeling. Second, individual participant data (IPD) meta-analyses or targeted trials in AD subgroups defined by disease stage and biomarker-based pathological subtypes could help identify optimal populations and timing for exercise interventions. Third, integrating multimodal data—such as neuroimaging and blood-based biomarkers—would allow dynamic monitoring of neuroplastic changes during exercise, offering deeper mechanistic insight into the origin and sustainability of cognitive benefits. Finally, in clinical practice, greater emphasis should be placed on developing individualized and adaptable exercise prescriptions, along with systematic evaluation of adherence and safety in long-term interventions, to promote the standardized implementation of exercise as part of comprehensive AD management.

### 4.9 Hierarchical interpretation of evidence strength

Based on the findings of this meta-analysis and the GRADE assessment (29), we provide a hierarchical interpretation of evidence strength for each finding to ensure consistency across Methods, Results, and Conclusions:

1. Robust evidence (moderate certainty): The overall beneficial effect of exercise on cognitive function in AD patients (*g* = 0.19–0.22) reached statistical significance and is highly consistent with previous meta-analyses (73), supporting its clinical value as an adjunctive intervention in AD management. The robustness of this conclusion is limited by heterogeneity and publication bias, resulting in moderate certainty of evidence.
2. Preliminary evidence (low certainty): The moderating effects of exercise frequency and total weekly exercise duration reached statistical significance and partially explained between-study heterogeneity, providing preliminary support for dose–response relationships. However, given that these findings are based on univariate meta-regression analyses and considerable heterogeneity across original studies, they should be interpreted as hypothesis-generating evidence requiring confirmation through prospective trials (75).
3. Exploratory observations (very low certainty): The phase-specific association between total intervention duration and cognitive improvement emerged from restricted cubic spline modeling, but the overall moderating effect was not significant; thus, this should be interpreted as an exploratory observation. The non-significant findings for age and single-session duration are consistent with some previous studies (73), but limitations in the reporting of these variables across original studies should be noted.
4. Hypothesis-generating findings: The non-significant results of multivariate models indicate that the current data do not support precise exercise prescription recommendations based on multiple parameters. These findings provide directions for future research design rather than clinical guidance (32).

## 5 Conclusion

This systematic review aimed to synthesize the existing evidence regarding the impact of exercise on cognitive function in older adults patients with Alzheimer’s disease (AD). Through the analysis of 23 studies involving a total of 1,868 participants, the results demonstrated that exercise intervention can effectively alleviate the clinical symptoms of older adults AD patients. “Although the effects of exercise on core cognitive outcomes varied across studies, exercise overall holds promise as an intervention to alleviate symptoms in older adults with AD. This study further suggests that exercise frequency and intervention duration may exhibit nonlinear dose–response relationships with cognitive improvement, particularly during the early phase of intervention and under higher-frequency regimens. However, these findings should be regarded as exploratory and hypothesis-generating, warranting confirmation through well-designed prospective trials to establish optimal exercise prescription parameters”.

## Data Availability

The original contributions presented in the study are included in the article/Supplementary material, further inquiries can be directed to the corresponding author/s.

## Footnotes

1 Version 4.3.1; R Core Team, https://www.r-project.org/.
