# Supplementary material for: Effects of exercise on cognitive function in older patients with Alzheimer’s disease: a meta-regression and meta-analysis
Source: Front Public Health. 2026 Apr 8;14:1793973. doi: 10.3389/fpubh.2026.1793973 (PMC13099286; doi:10.3389/fpubh.2026.1793973)
Supplement: Supplementary file 1 [file Table_1.DOCX]

Content

[Appendix 1 Search strategy 2](#_Toc63700674)

[Appendix 2 Risk of bias 5](#_Toc63700678)

[Appendix 3 GRADE 7](#_Toc63700678)

## Appendix 1 Search strategy

Search Formulas from Eight Databases (Web of Science, PubMed, Embase, Cochrane, Ebsco, Scopus, SPORTDiscus, and PsycInfo)

| **PubMed** | |
| --- | --- |
| #1 | Alzheimer Disease[Title/Abstract] OR Dementia[Title/Abstract] OR Cognitive Dysfunction[Title/Abstract] OR Amyloid beta-Peptides[Title/Abstract] OR tau Proteins[Title/Abstract] OR Neurodegenerative Diseases[Title/Abstract] |
| #2 | exercise[Title/Abstract] OR physical activity[Title/Abstract] OR physical exercise[Title/Abstract] OR training[Title/Abstract] ORaerobic exercise[Title/Abstract] OR resistance training[Title/Abstract] OR strength training[Title/Abstract] OR balance training[Title/Abstract] OR motor activity[Title/Abstract] OR mind-body exercise[Title/Abstract] OR walking[Title/Abstract] OR jogging[Title/Abstract] OR running[Title/Abstract] OR cycling[Title/Abstract] OR swimming[Title/Abstract] OR yoga[Title/Abstract] OR Tai Chi[Title/Abstract] OR qigong[Title/Abstract] OR high-intensity interval training[Title/Abstract] OR HIIT[Title/Abstract] OR Dancing[Title/Abstract] |
| #3 | Randomized Controlled Trial[Title/Abstract] OR Controlled Clinical Trial[Title/Abstract] OR Random Allocation[Title/Abstract] OR Double-Blind Method[Title/Abstract] OR Single-Blind  Method[Title/Abstract] |
| #4 | #1 AND #2 AND #3 |
| **Web of Science** | |
| #1 | TS=("Alzheimer Disease") OR TS=("Dementia") OR TS=("Cognitive Dysfunction" |
| #2 | TS=("exercise") OR TS=("Exercise Therapy") OR TS=("Physical Exertion") OR TS=("Athletic Performance") OR TS=("Aerobic Exercise") OR TS=("Resistance Training") OR TS=("Strength Training") OR TS=("Tai Ji") OR TS=("Yoga") OR TS=("Walking") OR TS=("Running") OR TS=("Swimming") OR TS=("Dancing") OR TS=("Physical exercise") OR TS=("training") OR TS=("balance training") OR TS=("motor activity") OR TS=("mind-body exercise") OR TS=("jogging") OR TS=("cycling") OR TS=("taichi") OR TS=("qigong") OR TS=("high-intensity interval training") OR TS=("hint") |
| #3 | TS=("Randomized Controlled Trial") OR TS=("Controlled Clinical Trial") OR TS=("Random Allocation") OR TS=("Double-Blind Method") OR TS=("Single-Blind Method") |
| #4 | #1 AND #2 AND #3 |
| **Embase** | |
| #1 | 'alzheimer disease'/ab OR 'dementia'/ab OR 'cognitive dysfunction'/ab OR 'amyloid beta-peptides'/ab OR 'tau proteins'/ab OR 'neurodegenerative diseases'/ab |
| #2 | 'exercise'/ab OR 'physical activity'/ab OR 'physical exercise'/ab OR 'training'/ab OR 'aerobic exercise'/ab OR 'resistance training'/ab OR 'strength training'/ab OR 'balance training'/ab OR 'motor activity'/ab OR 'mind-body exercise'/ab OR 'walking'/ab OR 'jogging'/ab OR 'running'/ab OR 'cycling'/ab OR 'swimming'/ab OR 'yoga'/ab OR 'tai chi'/ab OR 'qigong'/ab OR 'high-intensity interval training'/ab OR 'hiit'/ab OR 'dancing'/ab |
| #3 | 'randomized controlled trial'/ab OR 'controlled clinical trial'/ab  OR 'random allocation'/ab OR 'double-blind method'/ab OR 'single-blind method'/ab |
| #4 | #1 AND #2 AND #3 |
| **Cochrane** | |
| #1 | Alzheimer Disease OR Dementia OR Cognitive Dysfunction |
| #2 | exercise OR exercise therapy OR physical exercise OR athletic  performance |
| #3 | aerobic exercise OR resistance training OR strength training OR tai ji OR balance training OR motor activity OR mind-body exercise OR walking OR jogging OR running OR cycling OR swimming OR yoga OR Tai Chi OR qigong OR high-intensity interval training OR HIIT OR Dancing |
| #4 | Randomized Controlled Trial OR Controlled Clinical Trial OR Random Allocation OR Double-Blind Method OR Single-Blind Method |
| #5 | #1 AND #2 AND #3 AND #4 |
| **Ebsco** | |
| #1 | "Alzheimer Disease" OR "Dementia" OR "Cognitive Dysfunction" OR "Amyloid beta-Peptides" OR "tau Proteins" OR "Neurodegenerative Diseases" |
| #2 | "Exercise" OR "Exercise Therapy" OR "Physical Exertion" OR "Athletic Performance" OR "Aerobic Exercise" OR "Resistance Training" OR "Strength Training" OR "Tai Ji" OR "Yoga" OR "Walking" OR "Running" OR "Swimming" OR "Dancing" |
| #3 | "Randomized Controlled Trial" OR "Controlled Clinical Trial" OR "Random Allocation" OR "Double-Blind Method" OR "Single-Blind Method" |
| #4 | #1 AND #2 AND #3 |
| **Scopus** | |
| #1 | TITLE-ABS(Alzheimer Disease) OR TITLE-ABS(Dementia) OR TITLE-ABS(Cognitive Dysfunction |
| #2 | TITLE-ABS-KEY(exercise) OR TITLE-ABS-KEY(exercise therapy) OR TITLE-ABS-KEY(physical exercise) OR TITLE-ABS-KEY(athletic performance) |
| #3 | TITLE-ABS-KEY(aerobic exercise) OR TITLE-ABS-KEY(resistance  training) OR TITLE-ABS-KEY(strength training) OR TITLE-ABS-KEY(tai ji) OR TITLE-ABS-KEY(balance training) OR TITLE-ABS-KEY(motor activity) OR TITLE-ABS-KEY(mind-body exercise) OR TITLE-ABS-KEY(walking) OR TITLE-ABS-KEY(jogging) OR TITLE-ABS-KEY(running) OR TITLE-ABS-KEY(cycling) OR TITLE-ABS-KEY(swimming) OR TITLE-ABS-KEY(yoga) OR TITLE-ABS-KEY(Tai Chi) OR TITLE-ABS-KEY(qigong) OR TITLE-ABS-KEY(high-intensity interval training) OR TITLE-ABS-KEY(HIIT) OR TITLE-ABS-KEY(Dancing) |
| #4 | TITLE-ABS-KEY(Randomized Controlled Trial) OR TITLE-ABS-KEY(Controlled Clinical Trial) OR TITLE-ABS-KEY(Random Allocation) OR TITLE-ABS-KEY(Double-Blind Method) OR TITLE-ABS-KEY(Single-Blind Method) |
| #5 | #1 AND #2 AND #3 AND #4 |
| **SPORTDiscus** | |
| #1 | DE "ALZHEIMER'S disease" OR DE "DEMENTIA" OR DE "COGNITIVE impairment" |
| #2 | DE "EXERCISE" OR DE "PHYSICAL activity" OR DE "AEROBIC exercises" OR DE "STRENGTH training" OR DE "BALANCE training" OR DE "MIND & body exercises" OR DE "WALKING" OR DE "RUNNING" OR DE "SWIMMING" OR DE "YOGA" OR DE "TAI chi" OR DE "QIGONG" OR DE "DANCE" |
| #3 | DE "RANDOMIZED controlled trials" OR DE "CLINICAL trials" |
| #4 | #1 AND #2 AND #3 |
| **PsycInfo** | |
| #1 | DE "Alzheimer's Disease" OR DE "Dementia" OR DE "Cognitive Impairment" |
| #2 | DE "Exercise" OR DE "Physical Activity" OR DE "Aerobic Exercise" OR DE "Strength Training" OR DE "Balance Training" OR DE "Yoga" OR DE "Tai Chi" OR DE "Walking" OR DE "Running" OR DE "Swimming" OR DE "Dance" |
| #3 | DE "Randomized Controlled Trials" OR DE "Clinical Trials" |
| #4 | #1 AND #2 AND #3 |

## Appendix 2 Risk of bias

Table 2 Risk of bias assessment

| **study** | **D1** | **D2** | **D3** | **D4** | **D5** | **overall** |
| --- | --- | --- | --- | --- | --- | --- |
| Swinnen et al.2021 | Low | Low | Low | Low | Low | Low |
| Arcoverde et al.2013 | Some concerns | Low | Low | Low | Low | Some concerns |
| PapatsimpasAerobic at al.2023 | Some concerns | Low | Low | Low | Some concerns | Some concerns |
| Yang at al.2015 | Some concerns | Some concerns | Low | Some concerns | Some concerns | Some concerns |
| Enette at al.2020 | Some concerns | Low | Low | Low | Low | Some concerns |
| Angiolillo at al.2023 | Some concerns | Low | Some concerns | Low | Some concerns | Some concerns |
| David at at al.2025 | Low | Low | Low | Low | Low | Low |
| CavalcanteRE at al.2020 | Low | Low | Low | Low | Low | Low |
| Hoffmann at al.2016 | Low | Low | Low | Low | Low | Low |
| Fang Yu et al.2021 | Low | Low | Low | Low | Low | Low |
| Morrisat at al.2017 | Low | Low | Low | Low | Low | Low |
| Vreugdenhil et al.2011 | Some concerns | Low | Low | Low | Some concerns | Low |
| Eggermont et al. 2009 | High | Low | Low | Low | Low | Low |
| Henskens et al.2018 | Low | Low | Low | Low | Low | Low |
| Huang at al.2019 | Low | Some concerns | Low | Some concerns | Low | Low |
| Kemoun at al.2019 | Some concerns | Low | High | Some concerns | Some concerns | High |
| Lamb at al.2018 | Low | Low | Low | Low | Low | Low |
| Parvin at al.2020 | Some concerns | Low | Some concerns | Low | Low | High |
| Shaw at al.2021 | Some concerns | Some concerns | High | Some concerns | Some concerns | High |
| Sobol at al.2018 | Low | Low | Low | Low | Low | Low |
| Winckel at al.2004 | Some concerns | Low | High | Low | Some concerns | High |
| Toots at al.2017 | Low | Low | Low | Low | Low | Low |
| Christofoletti at al.2007 | Some concerns | Low | High | Low | Some concerns | High |


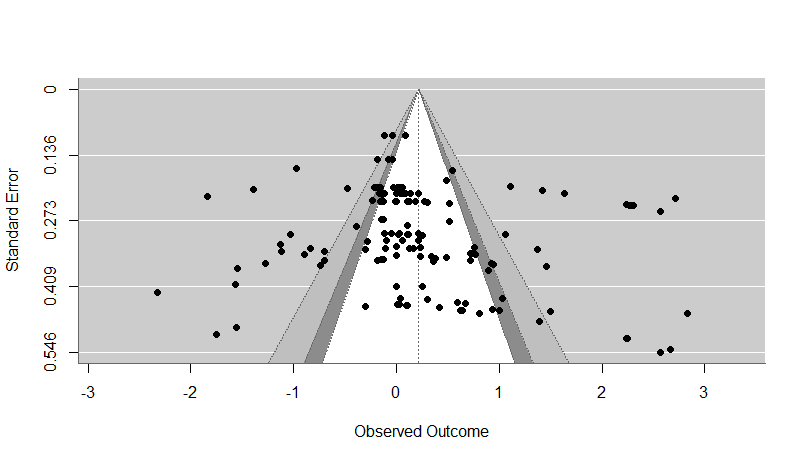


**Appendix 3 GRADE**

**Table 1** GRADE certainty of evidence assessments (main effect)

| **Certainty assessment** | | | | | | | **№ of patients** | | **Effect** | | **Certainty** | **Importance** |
| --- | --- | --- | --- | --- | --- | --- | --- | --- | --- | --- | --- | --- |
| **№ of studies** | **Study design** | **Risk of bias** | **Inconsistency** | **Indirectness** | **Imprecision** | **Other considerations** | **Exercise** | **Control** | **Relative**  **(95% CI)** | **Absolute**  **(95% CI)** |  |  |
| 144 | randomised trials | not serious | serious^b^ | not serious | not serious | publication bias strongly suspected^d^; | 1051 | 798 | - | SMD **0.22** (0.02 to 0.41) | ⨁⨁◯◯ Low | IMPORTANT |

**CI:** confidence interval; **SMD:** standardised mean difference

#### Explanations

a. Substantial heterogeneity was present in the primary analysis (*I*^2^ = 55%).

d. Egger’s test indicated publication bias (intercept = 0.87, *p=*0.01), and removal of five outliers from the primary analysis resulted in a reduction in the effect size estimate from *g* = 0.22(moderate) to *g* = 0.19 (small).

**Table 2** GRADE certainty of evidence assessments (subgroup analysis – effect of age on exercise interventions)

| **Certainty assessment** | | | | | | | **№ of patients** | | **Effect** | | **Certainty** | **Importance** |
| --- | --- | --- | --- | --- | --- | --- | --- | --- | --- | --- | --- | --- |
| **№ of studies** | **Study design** | **Risk of bias** | **Inconsistency** | **Indirectness** | **Imprecision** | **Other considerations** | **Exercise** | **Control** | **Relative (95% CI)** | **Absolute (95% CI)** |  |  |
| **≤75 year** | | | | | | | | | | | | |
| 46 | randomised trials | not serious | serious^a^ | not serious | not serious | no publication bias suspected | 265 | 254 | - | SMD **0.46**  (0.18 to 0.73) | ⨁⨁◯◯ Low | IMPORTANT |
| **＞75 year** | | | | | | | | | | | | |
| 97 | randomised trials | not serious | serious^b^ | not serious | serious^c^ | no publication bias suspected | 771 | 413 | - | SMD **0.10**  (-0.14 to 0.34) | ⨁◯◯◯ Very low | IMPORTANT |

**CI:** confidence interval; **SMD:** standardised mean difference

#### Explanations

a. Substantial heterogeneity was present in the primary analysis (*I*^2^ = 67.5%).

b. Substantial heterogeneity was present in the primary analysis (*I*^2^ = 76.4%).

c. 95% CI spans 0 with wide interval and small sample size(SMD 0.10(-0.14 to 0.34)).

**Table 3** GRADE certainty of evidence assessments (subgroup analysis – effect of frequency on exercise interventions)

| **Certainty assessment** | | | | | | | | | | | | | **№ of patients** | | | | **Effect** | | | | **Certainty** | **Importance** |
| --- | --- | --- | --- | --- | --- | --- | --- | --- | --- | --- | --- | --- | --- | --- | --- | --- | --- | --- | --- | --- | --- | --- |
| **№ of studies** | **Study design** | | **Risk of bias** | | **Inconsistency** | | **Indirectness** | | **Imprecision** | | **Other considerations** | | **Exercise** | | **Control** | | **Relative (95% CI)** | | **Absolute (95% CI)** | |  |  |
| **≤3 times/week** | | | | | | | | | | | | | | | | | | | | | | |
| 54 | | randomised trials | | not serious | | serious^a^ | | not serious | | serious^c^ | | no publication bias suspected | | 480 | | 241 | | - | | SMD **0.27**  (-0.06 to 0.60) | ⨁◯◯◯ Very low | IMPORTANT |
| **3-5 times/week** | | | | | | | | | | | | | | | | | | | | | | |
| 60 | | randomised trials | | not serious | | not serious | | not serious | | serious^d^ | | no publication bias suspected | | 404 | | 361 | | - | | SMD **0.06**  (-0.19 to 0.31) | ⨁⨁⨁◯ Moderate | IMPORTANT |
| **＞5 times/week** | | | | | | | | | | | | | | | | | | | | | | |
| 29 | | randomised trials | | not serious | | serious^b^ | | not serious | | not serious | | no publication bias suspected | | 92 | | 87 | | - | | SMD **0.54**  (0.12 to 0.96) | ⨁⨁◯◯ Low | IMPORTANT |

**CI:** confidence interval; **SMD:** standardised mean difference

#### Explanations

a. Substantial heterogeneity was present in the primary analysis (*I*^2^ = 86.06%).

b. Substantial heterogeneity was present in the primary analysis (*I*^2^ = 88.6%).

d. 95% CI spans 0 with wide interval and small sample size(SMD 0.27(-0.06 to 0.60)).

e. 95% CI spans 0 with wide interval and small sample size(SMD 0.06(-0.19 to 0.31)).

**Table 4** GRADE certainty of evidence assessments (subgroup analysis – effect of Single exercise duration on Exercise interventions)

| **Certainty assessment** | | | | | | | **№ of patients** | | **Effect** | | **Certainty** | **Importance** |
| --- | --- | --- | --- | --- | --- | --- | --- | --- | --- | --- | --- | --- |
| **№ of studies** | **Study design** | **Risk of bias** | **Inconsistency** | **Indirectness** | **Imprecision** | **Other considerations** | **Exercise** | **Control** | **Relative (95% CI)** | **Absolute (95% CI)** |  |  |
| **≤30 min** | | | | | | | | | | | | |
| 57 | randomised trials | not serious | serious^a^ | not serious | serious^d^ | no publication bias suspected | 128 | 132 | - | SMD **0.05** (-0.32 to 0.42) | ⨁◯◯◯ Very low | IMPORTANT |
| **30-60 min** | | | | | | | | | | | | |
| 45 | randomised trials | not serious | serious^b^ | not serious | serious^e^ | no publication bias suspected | 319 | 282 | - | SMD **0.12**  (-0.19 to 0.43) | ⨁⨁◯◯ Low | IMPORTANT |
| **≥60 min** | | | | | | | | | | | | |
| 41 | randomised trials | not serious | serious^c^ | not serious | not serious | publication bias strongly suspected^d^ | 572 | 371 | - | SMD **0.47**  (0.13to 0.80) | ⨁⨁⨁◯ Moderate | IMPORTANT |

**CI:** confidence interval; **SMD:** standardised mean difference

#### Explanations

a. Substantial heterogeneity was present in the primary analysis (*I*^2^ = 83%).

b.Substantial heterogeneity was present in the primary analysis (*I*^2^ = 43%)

c.Substantial heterogeneity was present in the primary analysis (*I*^2^ = 39.1%)

d. 95% CI spans 0 with wide interval and small sample size(SMD 0.05(-0.32 to 0.42)).

e. 95% CI spans 0 with wide interval and small sample size(SMD 0.12(-0.19 to 0.43)).

**Table 5** GRADE certainty of evidence assessments (subgroup analysis – effect of total interference duration on Exercise interventions)

| **Certainty assessment** | | | | | | | **№ of patients** | | **Effect** | | **Certainty** | **Importance** |
| --- | --- | --- | --- | --- | --- | --- | --- | --- | --- | --- | --- | --- |
| **№ of studies** | **Study design** | **Risk of bias** | **Inconsistency** | **Indirectness** | **Imprecision** | **Other considerations** | **Exercise** | **Control** | **Relative (95% CI)** | **Absolute (95% CI)** |  |  |
| **≤ 1 year** | | | | | | | | | | | | |
| 80 | randomised trials | not serious | not serious | not serious | not serious | publication bias strongly suspected^e^; large magnitude of effect^f^ | 771 | 413 | - | SMD **0.36**  (0.08 to 0.64) | ⨁⨁⨁⨁ High | IMPORTANT |
| **1-2 year** | | | | | | | | | | | | |
| 30 | randomised trials | not serious | serious^a^ | not serious | serious^c^ | no publication bias suspected | 489 | 280 | - | SMD **0.09**  (-0.19 to 0.37) | ⨁⨁◯◯ Low | IMPORTANT |
| **≥ 2 year** | | | | | | | | | | | | |
| 33 | randomised trials | not serious | serious^b^ | not serious | serious^d^ | no publication bias suspected | 416 | 340 | - | SMD **0.09**  (-0.21 to 0.40) | ⨁◯◯◯ Very low | IMPORTANT |

**CI:** confidence interval; **SMD:** standardised mean difference

#### Explanations

a. Substantial heterogeneity was present in the primary analysis (*I*^2^ = 75.48%).

c. Substantial heterogeneity was present in the primary analysis (*I*^2^ = 91.02%).

d. 95% CI spans 0 with wide interval and small sample size(SMD 0.09(-0.19 to 0.37)).

f. 95% CI spans 0 with wide interval and small sample size(SMD 0.09(-0.21 to 0.40)).

**Table 6** GRADE certainty of evidence assessments (subgroup analysis – effect of weekly exercise duration on exercise interventions)

| **Certainty assessment** | | | | | | | **№ of patients** | | **Effect** | | **Certainty** | **Importance** |
| --- | --- | --- | --- | --- | --- | --- | --- | --- | --- | --- | --- | --- |
| **№ of studies** | **Study design** | **Risk of bias** | **Inconsistency** | **Indirectness** | **Imprecision** | **Other considerations** | **Exercise** | **Control** | **Relative (95% CI)** | **Absolute (95% CI)** |  |  |
| **＜90 min** | | | | | | | | | | | | |
| 60 | randomised trials | not serious | serious^a^ | not serious | not serious | no publication bias suspected | 489 | 280 | - | SMD **0.10**  (-0.25 to 0.44) | ⨁⨁⨁◯ Moderate | IMPORTANT |
| **≥ 90 min** | | | | | | | | | | | | |
| 83 | randomised trials | not serious | not serious | not serious | serious^b^ | no publication bias suspected | 771 | 413 | - | SMD **0.27**  (0.04 to 0.51) | ⨁⨁◯◯ Low | IMPORTANT |

**CI:** confidence interval; **SMD:** standardised mean difference

#### Explanations

a. 95% CI spans 0 with wide interval and small sample size(SMD 0.10(-0.25 to 0.44)).

b. Substantial heterogeneity was present in the primary analysis (*I*^2^ = 74%
